# Natural compounds protect against the pathogenesis of osteoarthritis by mediating the NRF2/ARE signaling

**DOI:** 10.3389/fphar.2023.1188215

**Published:** 2023-05-30

**Authors:** Zhenyu Wu, Zhouxin Yang, Luying Liu, Yong Xiao

**Affiliations:** ^1^ First Affiliated Hospital of Gannan Medical University, Ganzhou, China; ^2^ First Clinical Medical College of Gannan Medical University, Ganzhou, China; ^3^ Jiangxi University of Traditional Chinese Medicine, Nanchang, China; ^4^ Xiaoyong Traditional Chinese Medicine Clinic in Yudu, Ganzhou, China

**Keywords:** osteoarthritis, oxidative stress, Nrf2/ARE signaling, flavonoids, terpenoids

## Abstract

Osteoarthritis (OA), a chronic joint cartilage disease, is characterized by the imbalanced homeostasis between anabolism and catabolism. Oxidative stress contributes to inflammatory responses, extracellular matrix (ECM) degradation, and chondrocyte apoptosis and promotes the pathogenesis of OA. Nuclear factor erythroid 2-related factor 2 (NRF2) is a central regulator of intracellular redox homeostasis. Activation of the NRF2/ARE signaling may effectively suppress oxidative stress, attenuate ECM degradation, and inhibit chondrocyte apoptosis. Increasing evidence suggests that the NRF2/ARE signaling has become a potential target for the therapeutic management of OA. Natural compounds, such as polyphenols and terpenoids, have been explored to protect against OA cartilage degeneration by activating the NRF2/ARE pathway. Specifically, flavonoids may function as NRF2 activators and exhibit chondroprotective activity. In conclusion, natural compounds provide rich resources to explore the therapeutic management of OA by activating NRF2/ARE signaling.

## Introduction

Osteoarthritis (OA), a degenerative and irreversible joint disease, has been associated with chronic inflammation, trauma, and other diseases. No effective strategies are available to cure OA. Clinically, non-steroidal anti-inflammatory drugs (NSAIDs) have been used to alleviate OA symptoms, such as joint pain, stiffness, and movement limitation, which may greatly decrease the quality of life and increase the burden on society and patients (Lane et al., 2017). This awkward situation might be attributed to the incomplete understanding of the pathological molecular mechanisms. It is well-accepted that OA is correlated with the imbalance between anabolism and catabolism in the joint cartilage. Both the reduction of anabolism and the stimulation of catabolism can be driven by chronic inflammatory responses, oxidative stress, and other aberrant signaling pathways. Effective agents used for preventing or inhibiting OA progression are still lacking.

Excessive oxidative stress contributes to the pathological development of OA (Lepetsos and Papavassiliou, 2016). Reactive oxygen/nitrogen species (ROS/RNS), such as hydrogen peroxide (H_2_O_2_), hydroxyl radical, superoxide anion, nitric oxide (NO), and hypochlorite ion, are free radicals, which have unpaired electrons. ROS can be produced by mitochondrion, non-mitochondrial membrane-bound nicotinamide adenine dinucleotide phosphate (NADPH) oxidases (NOXs), and xanthine oxidase (XO) (Turrens, 2003). Overproduction of ROS may induce damage to macromolecules, including DNA, protein, and fat. In addition, ROS can be the player involved in the intracellular signaling pathways, which orchestrate the homeostasis of joint cartilage. Increasing evidence shows that ROS overproduction and oxidative stress have been observed in the joint cartilage of patients with OA, and oxidative stress has been implicated in the mediation of chondrocyte apoptosis and extracellular matrix (ECM) degradation (Henrotin et al., 1993; Ertürk et al., 2012; Altay et al., 2015). Thus, suppression of oxidative stress has become a therapeutic strategy for OA management.

Nuclear factor erythroid 2-related factor 2 (NRF2), a transcriptional factor, exhibits a central role in maintaining the homeostasis of the intracellular redox system. NRF2 activation has been reported to counteract ROS generation and oxidative stress by binding to antioxidant response elements (AREs) in the promoters of the downstream antioxidant genes, such as heme oxygenase-1 (HO-1) (Baird and Yamamoto, 2020). Potentially, any exogenous bioactive ROS-scavenging or NRF2/ARE-activating agents that balance the redox homeostasis may lead to therapeutic effects against OA development. Naturally occurring compounds from different sources have been explored to develop novel preventive and therapeutic agents for treating various human diseases. Many of them may regulate multiple signaling pathways at DNA, RNA, and protein levels and exhibit favorable safety profiles (Khan et al., 2021). These make them become the ideal options for the therapeutic management of chronic diseases, such as OA. Natural compounds with anti-oxidative activity, such as flavonoids, have been linked to the confrontation of OA progression by inhibiting oxidative stress in chondrocytes (Wang Q. et al., 2022; Zhu et al., 2022a). In this article, we will summarize the protective activities of natural compounds against OA development by mediating the NRF2/ARE signaling pathway in chondrocytes.

## The interaction between oxidative stress and OA

### The pathological development of OA

OA elucidates a subset of the pathological alterations that are influencing the joints. The pathogenesis of OA includes three overlapping stages (Braaten et al., 2022). Firstly, ECM on the surface of the articular cartilage is damaged. Various factors, such as genetic, metabolic, biochemical, and environmental processes, contribute to the destruction of ECM (Felson et al., 2000; Loeser, 2010). Progressive ECM degeneration may lead to the development of clefts and fibrillation in the articular cartilage. Particularly, these pathological changes may affect the micro-environment of subchondral bone, leading to alterations in joint shape and loading transmission. Secondly, chondrocytes, the unique cell type in the joint cartilage, are affected by the damage of ECM and subchondral tissues. Importantly, many signaling pathways regulating the repair activity, such as bone morphogenic proteins (BMPs), transforming growth factor-beta (TGF-β), and insulin-like growth factor-I (IGF-I), are activated. On the other hand, ECM breakdown may trigger the release of pro-inflammatory cytokines and facilitate the expression of matrix metalloproteases (MMPs), forming a vicious cycle of cartilage degeneration. In addition, endochondral ossification is also stimulated by up-regulating the expression of type X collagen. The third stage is the failure of cartilage repair during OA development. The repair activity of chondrocytes is rather limited. The continued catabolic activity progressively contributes to the damage to cartilage and subchondral bone and cell death. For example, upregulated expression of MMPs and downregulated expression of tissue inhibitors of metalloproteinase-1 (TIMP-1) are reported in OA chondrocytes (Lotz and Loeser, 2012).

The pathological changes of OA are associated with the dysregulation of signaling pathways, such as NRF2/ARE, NF-κB, AMPK/Sirt1, HIFs, Wnt/β-catenin, TGFβ/BMP, and JAK2/STAT3 (Park and Lee, 2022; Yao et al., 2023). NRF2/ARE signaling is a major factor in counteracting oxidative stress and ECM degradation. Activation of the NRF2 signaling facilitates to maintain the homeostasis of chondrocytes and joint cartilage (Xiong et al., 2023). In addition, activation of the NRF2 signaling may negatively regulate the phosphorylation and nuclear translocation of p65, suppressing the NF-κB and RANKL signaling pathways in IL-1β-treated chondrocytes (Yang et al., 2023). HIF-1α exhibits protective activity on articular cartilage by suppressing the NF-κB signaling in mice. Loss of HIF-1α expression upregulates the expression of MMP-13 and HIF-2α (Okada et al., 2020). Dysregulation of the Wnt/β-catenin signaling contributes to the pathological development of OA. Increased expression of *β*-catenin has been found in OA patients, and genetic activation of *β*-catenin stimulates the expression of catabolic enzymes, such as MMPs and ADAMTSs (Hui et al., 2018). Similarly, the TGFβ/BMP/Smad signaling pathway also plays a key role in cartilage homeostasis. Smad2/3 and Smad1/5/9 may exhibit distinctive effects on chondrocyte biology. In chondrocyte-specific Smad3-null mice, dysregulated expression of MMP-13 and Col10a1 is found, and increased chondrocyte hypertrophy and stimulated cartilage degeneration are observed (Chen et al., 2012). Combined knockout of Smad1, 5, and 8 may induce severe chondrodysplasia (Retting et al., 2009). The JAK2/STAT3 signaling is abnormally activated in OA chondrocytes. Inhibition of JAK2 expression can lead to a significant reduction in aggrecan loss and chondrocyte apoptosis (Lu et al., 2017). Targeting the JAK2/STAT3 signaling has become a potential therapeutic approach for OA treatment (Chen et al., 2023). Thus, the pathological development of OA is related to multi-factors and multi-pathways. Studies have shown that these signaling pathways can be potential targets for the therapeutic management of OA (Cheng et al., 2022; van der Kraan, 2022).

### The involvement of oxidative stress in the pathogenesis of OA

Oxidative stress contributes to inflammatory responses in OA chondrocytes. Inflammatory responses are involved in the dysregulated balance between anabolism and catabolism in chondrocytes (Scanzello and Goldring, 2012). The balance between PI3K/AKT and MAPK signaling pathways exhibits a critical role in inflammatory responses and OA pathogenesis (Yu and Kim, 2015). Increased ROS production in OA chondrocytes has been demonstrated to suppress the activity of the PI3K/AKT pathway. In addition, ROS may stimulate the expression of the MAPK pathway. Thymoquinone (TQ), a major metabolite of black seed oil, can induce ROS generation. TQ may increase the production of COX-2 and PGE2 by activating the p38 and ERK pathways in rabbit OA chondrocytes. N-acetyl cysteine (NAC), an antioxidant, may abolish TQ-induced ROS production and inflammatory responses (Yu and Kim, 2015). Advanced oxidation protein products (AOPPs) can enhance the expression of IL-1β and TNFα by up-regulating the p38-MAPK signaling in OA chondrocytes (Liao et al., 2020). H_2_O_2_ increases the secretion of IL-6 and TNFα by activating the NF-κB signaling in OA chondrocytes (Wang et al., 2023).

Oxidative stress facilitates ECM degradation. NOX enzyme family is a key source of superoxide anion and H_2_O_2_. NOX-4 may enhance IL-1β-stimulated expression of MMPs, which promote ECM degradation (Grange et al., 2006). NOX4 deficiency decreases MMP-13 and collagen I expression, enhances aggrecan production, and reduces cartilage degradation in DMM-induced OA mice (Renaudin et al., 2023). Another study reports that AOPPs may upregulate the expression of NOX4 in chondrocytes. Apocynin, a NOX inhibitor, can block the detrimental effects of AOPPs, as shown by increased collagen II and GAG expression and attenuated ECM degradation (Liao et al., 2020). Sirt4 deficiency downregulates the expression of antioxidant enzymes, such as SOD1, SOD2, and CAT. Knockdown of Sirt4 may decrease the production of collagen II and aggrecan and increase the generation of MMP-13 in OA chondrocytes (Dai et al., 2020).

**FIGURE 1:**
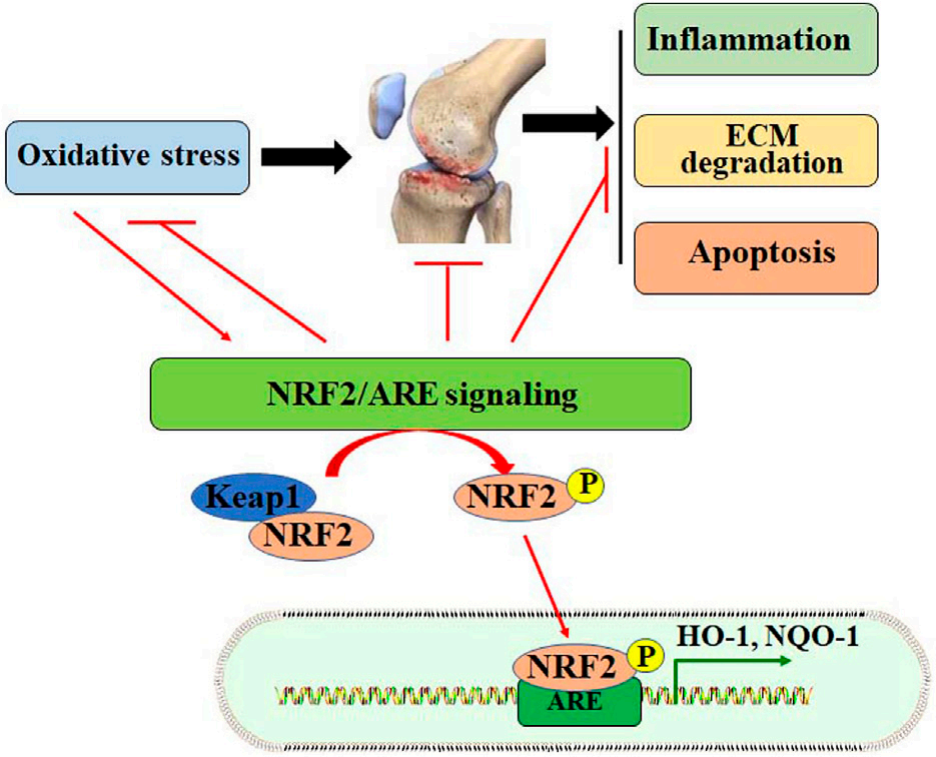
Oxidative stress is implicated in the pathogenesis of OA. Oxidative stress may contribute to the development of OA by increasing inflammatory responses, ECM degradation, and chondrocyte apoptosis. Oxidative stress also stimulates the NRF2/ARE signaling pathway by dissociating Keap1 from NRF2. Activated NRF2 enters the nucleus for transcriptional regulation by binding to ARE, up regulating the expression of HO-1 and NQO-1.

Oxidative stress promotes cell death in OA chondrocytes. ROS production increases with aging, due to the decline of mitochondrial functions (Chistiakov et al., 2014). In addition, excessive mechanical loading in chondrocytes may induce the generation of superoxide anion and decrease the expression of superoxide dismutase (SOD), which catalyzes the conversion of superoxide anion to H_2_O_2_ (Koike et al., 2015). Mitochondrial H_2_O_2_ can induce human chondrocyte cell death by up-regulating the activity of the MKK3/6-p38 signaling pathway (Collins et al., 2016). Similarly, ROS also promotes chondrocyte cell death by activating protein kinase C (PKC-β1) expression, and inhibition of PKC-β1 may abolish ROS-induced chondrocyte cell death (DelCarlo and Loeser, 2006). Advanced glycation end products (AGEs) may induce mitochondrial dysfunction in OA chondrocytes. It has been reported that AGEs can induce oxidative stress and chondrocyte apoptosis by down-regulating the AMPKα/Sirt1/PGC-1α signaling (Yang et al., 2022). Collectively, inhibiting redox-sensitive factors implicated in cell death and stimulating antioxidant expression may facilitate chondrocyte survival and promote cartilage integrity (Figure 1).

### The roles of the NRF2/ARE signaling in the pathological development of OA

It is well-accepted that the NRF2/ARE pathway is negatively mediated by Keap1 (Suraweera et al., 2020; Saha et al., 2021). Under normal conditions, NRF2-Keap1 complex interacts with the E3 ubiquitin ligase complex Cullin 3 (Cul3) and leads to the ubiquitination and proteasomal degradation of NRF2. Under stressed conditions, modification of the cysteine residues may induce the conformational changes of Keap1. Then, the conformational changes of Keap1 induce its dissociation from NRF2. The dissociation of Keap1 prevents NRF2 from ubiquitination and degradation, and NRF2 is stabilized and then translocated into the nucleus for transcriptional regulation (Dinkova-Kostova et al., 2018). Additionally, NRF2 can also be phosphorylated by PKC, casein kinase II (CKII), protein kinase R-like endoplasmic reticulum kinase (PERK), JNK, and ERK (Zuo et al., 2022).

Activation of the NRF2/ARE signaling pathway exhibits the protective activity against OA pathogenesis by up-regulating the expression of antioxidant factors, such as HO-1, NADPH gene of quinone oxidoreductase 1 (NQO1), GSH, GPx, and SOD, and suppressing oxidative stress in chondrocytes (Ashrafizadeh et al., 2020) (Figure 1). In OA chondrocytes, NRF2/ARE signaling activation is associated with anti-inflammatory effects. Specifically, activation of the NRF2/ARE signaling may suppress M1 polarization and promote M2 polarization through signaling transductions, including TGFβ/Smad, JAK/STAT, and NF-κB pathways (Wang and He, 2022). NLRP3 inflammasome can be stimulated by the TLR/NF-κB signaling pathway. It is also reported that activation of the NRF2 signaling may alleviate the progression of OA by suppressing NLRP3 inflammasome in primary mouse chondrocytes (Yan et al., 2020). MCC950 is an inhibitor of NLRP3. It has been shown that MCC950 may ameliorate inflammatory responses and protect cartilage against degeneration by up-regulating the expression of NRF2/HO-1/NQO-1 signaling pathway in mouse chondrocytes (Ni et al., 2021).

Activation of the NRF2 signaling also contributes to the inhibition of ECM degradation and chondrocyte apoptosis. It has been reported that NRF2 signaling may increase the expression of collagen II and SOX9 and decrease the expression of MMP-13 and ADAMTS5, protecting against IL-1β-induced ECM degradation in OA chondrocytes (Liang et al., 2022). Manganese (Mn) deficiency can negatively affect tibial cartilage development by stimulating oxidative stress and inflammation. Mn deficiency may downregulate NRF2 signaling and upregulate MMP-1, MMP-9, and MMP-13 expression in broiler chicks (Dong et al., 2022). Knockdown of NRF2 may promote MMP-13 expression, decrease aggrecan production, and suppress cell apoptosis in TBHP-treated chondrocytes (Shao et al., 2020). It has been demonstrated that NRF2 activation may suppress IL-1β-induced mitochondrial dysfunction, ROS generation, and apoptosis in OA chondrocytes. Mechanically, overexpression of NRF2 upregulates the expression of anti-apoptotic factors, downregulates pro-apoptotic proteins, and activates ERK1/2 and its downstream factors, such as ELK1, P70S6K, and P90RSK (Khan et al., 2018).

However, one study reports that inhibition of the NRF2/ARE signaling can ameliorate LPS-stimulated NLRP3 inflammasome in SW982 cells. Increased expression of NRF2/HO-1 and NLRP3 are also observed in rat OA models (Chen et al., 2019). Another study suggests that there is no difference in NRF2 expression between OA and healthy human cartilage chondrocytes (Cai et al., 2019b). The discrepancy in NRF2 expression in OA chondrocytes might be related to various factors, such as experimental conditions, cell types, and cell situations. Thus, more efforts are still needed for elucidating the roles of NRF2 in the pathological development of OA.

### Natural compounds activate the NRF2/ARE signaling to protect against OA development

**SCHEME 1:**
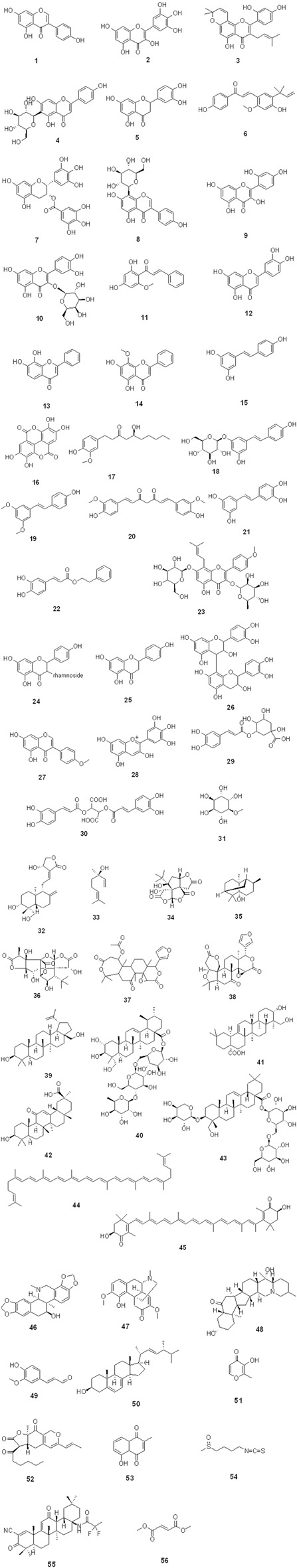
The chemical structures of natural compounds.

The important roles of oxidative stress in the development of OA indicate that suppression of oxidative stress can be a useful strategy for OA treatment. Exploration of natural compounds with anti-oxidative activity has become one of the research interests in treating redox-imbalanced diseases, such as OA. Dietary supplements and nutraceuticals are also involved in the therapeutic management of OA. For example, allicin, sulforaphane (SFN), and lycopene are the antioxidants isolated from garlic, broccoli, and tomato, respectively, and they may decrease oxidative stress and inflammatory responses. It has been reported that allicin, SFN, and lycopene can effectively inhibit H_2_O_2_-induced oxidative stress and chondrocytes apoptosis by activating the Keap1/NRF2 signaling pathway (Yang J. et al., 2020). Most natural compounds with anti-oxidative activity by activating the NRF2/ARE signaling pathway are mainly polyphenols and terpenoids (Scheme 1). Particularly, natural flavonoids may act as NRF2 activators to protect against oxidative stress.

### Polyphenols/flavonoids activate the NRF2 signaling

Flavonoids have various pharmacological activities, including anti-inflammation, anti-oxidation, anti-cancer, anti-apoptosis, and bone system protection (Ghitti et al., 2022). Natural flavonoids provide health-benefiting effects against chronic diseases, such as OA. Particularly, isoflavones can act as phytoestrogens due to their structural similarity to estrogen. The estrogen-like effects of flavonoids greatly trigger the research interest because they may provide similar effects to the hormones (Guo et al., 2022). Natural flavonoids can inhibit inflammatory responses, attenuate ECM degradation, and suppress cell death in OA chondrocytes.

### Inhibition of inflammatory responses

Genistein **1**) acts as a selective estrogen receptor modulator (SERM) due to its structural similarity to estrogen. It has been reported that genistein (10 μM) decreases the expression of COX-2, NOS2, and NO in IL-1β-treated chondrocytes by activating the NRF2 signaling (Liu et al., 2019). Myricetin **2**), extracted from Garlic (*Allium ampeloprasum* L., *Amaryllidaceae*) with the availability of 693 mg/kg (Agraharam et al., 2022), at the dose of 15 μM has been reported to decrease the expression of IL-6, TNFα, COX-2, PGE2, iNOS, and NO and inhibit the NF-κB signaling in IL-1β-treated chondrocytes by activating the NRF2 signaling (Pan et al., 2019). In addition, Moracin (**3**, isolated from *Cortex Mori.* Radicis, *Moraceae*), Isovitexin (**4**, isolated from the passion flower (*Passiflora edulis* Sims, *Passifloraceae*), Cannabis (*Cannabis sativa* L., *Moraceae*), and palm (*Elaeis guineensis* Jacq., *Arecaceae*)), Eriodictyol (**5**, isolated from citrus fruits (*Citrus reticulata* Blanco, *Rutaceae*)), Licochalcone A (Lico A, **6**, isolated from the licorice root (*Glycyrrhiza uralensis* Fisch., *Leguminosae*)), and Epigallocatechin-3-O-gallate (EGCG, **7**, isolated from tea leaves) exhibit similar effects against inflammatory responses by activating the NRF2 signaling in OA chondrocytes (Jia et al., 2017; Wang Y. et al., 2018; Zhou et al., 2020; Hu et al., 2021; Zhu et al., 2022a) (Tables 1, 2). In addition, EGCG (100 μM) also suppresses the JNK/MAPK signaling and inhibits inflammatory responses in IL-1β-treated human chondrocytes (Akhtar and Haqqi, 2011).

**TABLE 1:** The protective effects of some natural compounds against OA development by mediating the NRF2 signaling pathway in animal models.

Compounds	Sources and availability	Models	Doses	Routines	Biological actions	Ref
Genistein	Soybean; 26.8–102.5 mg/100 g	Rat ACLT	40 mg/kg/day	gavage	OARIS Score↓, GAG↑	Liu et al. (2019)
Myricetin	Garlic; 693 mg/kg	Mouse DMM	20 mg/kg/2 days	gavage	NRF2↑, p-Akt↑, OARIS Score↓	Pan et al. (2019)
moracin	*Cortex Mori.*; 0.01%–0.6%	Rat ACLT	30 mg/kg/day	gavage	OARIS Score↓, NRF2↑, collagen II↑	Zhou et al. (2020)
Lico A	licorice root; 4–10 mg/g	Mouse DMM	10 mg/kg/day	gavage	OARIS Score↓, NRF2↑, IL-1β↑, IL-18↑	Yan et al. (2020)
7,8-DHF	*Lepisorus ussuriensis*; 0.48 mg/kg	Mouse DMM	5 mg/kg/week	i.p	OARIS Score↓, NRF2↑, HO-1↑, IL-1β↓, IL-6↓, TNFα↓, MMP-1/-3/-13↓	Cai et al. (2019a)
Polydatin	cocoa powder; 7.14 μg/g	Mouse DMM	100 mg/kg/day	i.p	OARIS Score↓, Synovitis Score↓	Tang et al. (2018)
Engeletin	Grape skin; 2.4 mg/kg	Rat ACLT	50 μg/week	i.a	OARIS Score↓	Wang et al. (2021a)
18β-GA	*Glycyrrhiza glabra*; 0.1%–1.6%	Mouse DMM	50 mg/kg/day	i.p	OARIS Score↓, NRF2↑	Chen et al. (2021a)
ASD	Rhizome of *Dipsacus asper* Wall; 15.0 g/kg	Mouse DMM	100 mg/kg/3 days	gavage	OARIS Score↓, NRF2↑	Gu et al. (2020)
AST	*Haematococcus lacustris*; 4% of dry weight	Mouse DMM	20 mg/kg twice a week	i.a	OARIS Score↓, NRF2↑	Sun et al. (2019)
COR	*Corydalis bungeana Turcz.*; 9 mg/kg	Mouse DMM	15 and 30 mg/kg/day	gavage	OARIS Score↓, NRF2↑, collagen II↑	Li et al. (2022)

Note: i.p., intraperitoneal injections; i.a., intra-articular injection.

**TABLE 2:** The protective effects of some natural compounds against OA development by mediating the NRF2 signaling pathway in vitro

Compounds	Cell lines	Concentrations	Biological actions	Ref
moracin	Rat chondrocytes	5, 10, and 15 μM	NRF2↑, HO-1↑, p65 nuclear translocation↓, IL-6↓, TNFα↓, COX-2↓, PGE2↓, iNOS↓, NO↓, MMP-13↓, ADAMTS5↓	Zhou et al. (2020)
Lico A	Mouse chondrocytes	5, 25, and 50 μM	NRF2↑, HO-1↑, p65↓, NLRP3↓, Cleaved GSDMD↓, Cleaved caspse-1↓, collagen II↑, aggrecan↑	Yan et al. (2020)
Hyperoside	Mouse chondrocytes	10, 20, and 40 μM	ROS↓, COX-2↓, iNOS↓, MMP-3↓, MMP-13↓, ADAMTS5↓, collagen II↑, aggrecan↑, SOX9↑, cleaved caspase-3/-9↓, Bax↓, Bcl-xL↑, cytochrome c↓	Sun et al. (2021)
7,8-DHF	Mouse chondrocytes	1, 3, and 9 μM	NRF2↑, HO-1↑, MDA↓, SOD↑	Cai et al. (2019a)
Pterostilbene	Rat chondrocytes	4, 8, 10, and 20 μM	NRF2↑, COX-2↓, PGE2↓, iNOS↓, NO↓, ROS↓	Xue et al. (2017)
Engeletin	Rat chondrocytes	10 and 20 μM	NRF2↑, HO-1↑, NQO-1↑, P-p65↓, MAPK↓, MMP-3↓, MMP-9↓, collagen II↑, aggrecan↑	Wang et al. (2021a)
Biochanin A	Mouse chondrocytes	12 and 24 μM	MMP-3↓, collagen II↑, Bcl-2↑, TFR1↓, ROS↓, GPX4↑, xCT↑, NRF2↑, HO-1↑	He et al. (2022)
18β-GA	Mouse chondrocytes	10, 25, and 50 μM	NRF2↑, HO-1↑, IL-6↓, TNFα↓, COX-2↓, PGE2↓, iNOS↓, NO↓, MMP-13↓, ADAMTS5↓	Chen et al. (2021a)
ASD	Mouse chondrocytes	50, 100, and 200 μM	NRF2↑, HO-1↑, p65↓, IL-6↓, TNFα↓, COX-2↓, PGE2↓, iNOS↓, NO↓, MMP-13↓, ADAMTS5↓, collagen II↑, aggrecan↑	Gu et al. (2020)
AST	Mouse chondrocytes	5, 10, and 20 μM	NRF2↑, HO-1↑, p-p65/p65↓, MAPK↓, TNFα↓, COX-2↓, iNOS↓, MMP-3/-13↓, ADAMTS5↓, collagen II↑	Sun et al. (2019)
COR	Mouse chondrocytes	2 and 4 μM	NRF2↑, HO-1↑, p-p65/p65↓, IL-6↓, COX-2↓, iNOS↓, MMP-3/-13↓, ADAMTS5↓, collagen II↑, aggrecan↑	Li et al. (2022)

Puerarin (**8**), separated from the root of *Pueraria lobata* var. Lobata (Willd.) Ohwi (Leguminosae), at the doses of 100 mg/kg/day and 200 mg/kg/day for 14 days by gavage ameliorates the pathological changes of OA cartilage by activating AMPK and NRF2 signaling pathways in MIA-induced rat OA models (Wang L. et al., 2018). Additionally, puerarin (10, 25, and 50 μM) decreases the production of IL-6, TNFα, COX-2, PGE2, iNOS, and NO and inhibits the NF-κB signaling IL-1β-treated mouse chondrocytes (Chen X. et al., 2021). Morin (**9**, isolated from the Chinese botanical drugs *Ficus religiosa* L.) of the *Moraceae* family) decreases IL-1β-induced enhancement of NO and PGE_2_ production and activation of the NF-κB signaling in human chondrocytes. NRF2 siRNA transfection may abolish the suppressive activity of Morin against inflammatory responses (Qu et al., 2018) (Table 3). Hyperoside (**10**, isolated from *Epimedium brevicornu* Maxim (*Berberidaceae*), *Hypericum attenuatum* Fisch. (*Guttiferae*), and *Hypericum perforatum* L. (*Guttiferae*)) and Cardamonin (**11**, isolated from *Alpinia katsumadai* Hayata, *Zingiberaceae*) consistently protect mouse chondrocytes against IL-1β-induced COX-2 and iNOS expression by suppressing PI3K/AKT/NF-κB and MAPK signaling pathways via activating the NRF2 signaling (Peng et al., 2021; Sun et al., 2021) (**Table 2**). Luteolin (**12**) and 7,8-dihydroxyflavone (7,8-DHF, **13**) inhibit H_2_O_2_-induced inflammatory responses in mouse chondrocytes by inhibiting the MAPK pathway and activating the NRF2 signaling (Cai et al., 2019a; Zhou et al., 2022) (Tables 1, 2). Wogonin (5,7-dihydroxy-8-methoxyflavone, **14**), isolated from *Scutellaria baicalensis* Georgi (*Lamiaceae*), at the doses of 10, 25, and 50 μM has been reported the anti-inflammatory effects, which are not associated with the inhibition of NF-κB and MAPK signaling pathways in IL-1β-treated human chondrocytes (Khan et al., 2017) (Table 3).

**TABLE 3:** The clinical studies of natural compounds against OA development by mediating the NRF2/ARE signaling pathway.

Compounds	Models/Doses	Biological actions	Conclusions	Ref
Genistein	IL-1β-treated human chondrocytes/10 μM	NRF2↑, HO-1↑, COX-2↓, iNOS↓, MMP-1↓, MMP-3↓, MMP-13↓, collagen II↑, aggrecan↑	Inhibits inflammation and ECM degradation by activating the NRF2/ARE signaling	Liu et al. (2019)
Myricetin	IL-1β-treated human chondrocytes/5, 10, and 15 μM	NRF2↑, HO-1↑, p65↓, IL-6↓, TNFα↓, COX-2↓, PGE2↓, iNOS↓, NO↓, MMP-13↓, ADAMTS5↓, collagen II↑, aggrecan↑	Inhibits inflammation and ECM degradation by activating the NRF2/ARE signaling	Pan et al. (2019)
Morin	IL-1β-treated human OA chondrocytes/2.5, 5, and 10 μM	PGE2↓, NO↓, p65↓, NRF2↑, HO-1↑, MMP-1↓, MMP-3↓, MMP-13↓	Inhibits inflammation and ECM degradation by activating the NRF2/ARE signaling	Qu et al. (2018)
Wogonin	IL-1β-treated human OA chondrocytes/10, 25, and 50 μM	IL-6↓, TNFα↓, COX-2↓, PGE2↓, iNOS↓, NO↓, MMP-3↓, MMP-9↓, MMP-13↓, ADAMTS5↓, collagen II↑, ACAN↑, NRF2↑, HO-1↑, NQO-1↑, p-ERK1/2↑	Inhibits inflammation and ECM degradation by activating the NRF2/ARE signaling	Khan et al. (2017)
Polydatin	IL-1β-treated human chondrocytes/25, 50, and 100 μg/mL	NRF2↑, HO-1↑, IL-6↓, TNFα↓, COX-2↓, PGE2↓, iNOS↓, NO↓, MMP-13↓	Inhibits inflammation and ECM degradation by activating the NRF2/ARE signaling	Tang et al. (2018)
Curcumin	IL-1β-treated human chondrocytes/20 and 40 μM	IL-6↓, TNFα↓, COX-2↓, iNOS↓, MMP-1↓, MMP-3↓, MMP-9↓, MMP-13↓, ADAMTS4↓, ADAMTS5↓, COL2A1↑, ACAN↑, NRF2↑, HO-1↑, NQO-1↑, GCLC↑, SOD-2↑	Inhibits inflammation and ECM degradation by activating the NRF2/ARE signaling	Jiang et al. (2020a)
Piceatannol	IL-1β-treated human chondrocytes/1, 5, and 10 μM	IL-6↓, TNFα↓, COX-2↓, PGE2↓, iNOS↓, NO↓, MMP-13↓, ADAMTS5↓, collagen II↑, aggrecan↑, p65↓, NRF2↑, HO-1	Inhibits inflammation and ECM degradation by activating the NRF2/ARE signaling	Tang et al. (2017)
ICA	IL-1β-treated HC-A/10^−9^M	ROS↓, GPX↑, SOD↑, MMP-3↓, MMP-9↓, MMP-13↓, ADAMTS4↓, GAG↑, NRF2↑, NQO-1↑	Inhibits oxidative stress and ECM degradation by activating the NRF2/ARE signaling	Zuo et al. (2019)
CA	TNFα-treated C28/I2 cells/5, 10, and 20 μM	IL-1β↓, IL-6↓, IL-12↓, COX-2↓, PGE2↓, iNOS↓, NO↓, MMP-3↓, MMP-9↓, MMP-13↓, ADAMTS5↓, collagen II↑, aggrecan↑, ROS↓, p-PI3K↓, p-AKT↓, p65↓	Inhibits inflammation, oxidative stress, and ECM degradation by activating the NRF2/ARE signaling	Qu et al. (2022)
Hederagenin	IL-1β-treated C28/I2 cells/5, 10, and 20 μM	IL-6↓, TNFα↓, COX-2↓, PGE2↓, iNOS↓, NO↓, ROS↓, MMP-1↓, MMP-3↓, MMP-9↓, MMP-13↓, ADAMTS5↓, collagen II↑, aggrecan↑, Bax↓, Bcl-2↑, cleaved caspase-3↓, p-JAK2↓, p-STAT3↓, MAPK↓, NRF2↑	Inhibits inflammation, oxidative stress, chondrocyte apoptosis, and ECM degradation by activating the NRF2/ARE signaling	Shen et al. (2022)
SFN	H_2_O_2_-treated human OA chondrocytes/7 μM	NRF2↑, NOX4↓, COL2↑, ACAN↑, SOX9↑, IL-6↓, TNFα↓, MMP-13↓, ADAMTS5↓	Inhibits inflammation, oxidative stress, and ECM degradation by activating the NRF2/ARE signaling	Yang et al. (2020a)

Resveratrol (**15**), firstly extracted from the root of *Veratrum grandiflorum* O. Loes (*Liliaceae*), has been demonstrated to be an activator of Sirt1, which may mediate Wnt/β-catenin and Foxo1 signaling pathways in OA chondrocytes (Liang et al., 2023). Resveratrol at the dose of 50 mg/kg/3 days for 8 weeks can inhibit oxidative stress and suppress inflammatory responses in MIA-induced rat OA models by triggering the NRF2/HO-1 signaling and inhibiting the NF-κB pathway (Wei et al., 2018). TLR4/NF-κB/STAT3 and JAK2/STAT3 signaling pathways are also implicated in OA development, and they can be inhibited by Resveratrol (Limagne et al., 2016; Jiang M. et al., 2020). Ellagic acid (EA, **16**), a natural polyphenol from fruits and nuts, has anti-inflammatory and anti-oxidative activities. EA (10 and 25 μM) has been demonstrated to inhibit IL-1β-induced oxidative stress by activating the NRF2/HO-1-NQO-1 signaling pathway in C28/I2 chondrocytes (Zhu et al., 2022b). 6-Gingerol (**17**) at the doses of 2, 5 and 10 μM exhibits protective activity against 4-hydroxynonenal-induced chondrocyte cell death by activating the NRF2 signaling pathway. In addition, 6-gingerol suppresses inflammatory responses and oxidative stress. However, IL-1β-induced activation of MAPK and NF-κB is not mediated by 6-gingerol (Abusarah et al., 2017). Other polyphenols, such as Polydatin (**18**, extracted from the roots of *Polygonum cuspidatum* Sieb. Et Zucc, *Polygonaceae*), Pterostilbene (**19**, derived from berries), Curcumin (**20**, a major turmeric component), piceatannol (**21**, derived from the seeds of *Euphorbia lagascae* Spreng, *Euphorbiaceae*), and Caffeic acid phenethyl ester (CAPE, **22**, one of the major bioactive ingredients of propolis), also exhibit inhibitory activities against inflammatory responses by activating the NRF2 signaling pathway in IL-1β-treated chondrocytes (Tang et al., 2017; Xue et al., 2017; Tang et al., 2018; Jiang C. et al., 2020; Sun et al., 2022).

### Attenuation of ECM degradation

Myricetin (**2**), Moracin (**3**), and Puerarin (**8**) can protect against IL-1β-induced upregulation of MMP-13 and ADAMTS5 expression and downregulation of collagen II and aggrecan expression. Knockdown of NRF2 ameliorates the inhibitory effects of myricetin, moracin, and Puerarin on IL-1β-induced ECM degradation, respectively (Pan et al., 2019; Zhou et al., 2020; Chen X. et al., 2021) (Table 1). Hyperoside (**10**) and EGCG (**7**) inhibit IL-1β-induced ECM degradation by up-regulating collagen II, aggrecan, and SOX9 expression and down-regulating MMP-3, MMP-13, and ADAMTS5 expression via the activation of NRF2 signaling in mouse chondrocytes (Sun et al., 2021; Zhu et al., 2022a) (Table 2). Icariin (ICA, **23**, isolated from *herba epimedium* L. (*Berberidaceae*)) and Wogonin (**14**, derived from the root extract of *Scutellaria baicalensis* Georgi, *Lamiaceae*) may alleviate ECM degradation by activating the NRF2 signaling in IL-1β-treated human chondrocytes, as shown by decreased generation of ROS, downregulated expression of MMP-3, MMP-9, MMP-13, and ADAMTS-4, and increased expression of GAG (Zuo et al., 2019). Curcumin (**20**) promotes the expression of Col2α, aggrecan, and SOX9, suppresses ECM degradation, and increases chondrocyte viability by inhibiting the NF-κB/HIF-2α signaling (Wang P. et al., 2021).

Similarly, Lico A (**6**) suppresses the expression of MMP-1, MMP-13, and ADAMTS4/5 and enhances the expression of collagen II by inhibiting NF-κB and Wnt/β-catenin signaling pathways in rat chondrocytes (Chen et al., 2017). Isovitexin (**4**) at the doses of 25 and 50 μg/mL inhibits MMP-3, MMP-13, and ADAMTS5 expression and increases collagen II and aggrecan expression by activating the NFR2 signaling in IL-1β-treated mouse chondrocytes (Hu et al., 2021). Pterostilbene (**19**) at the doses of 10 and 20 μM also inhibits IL-1β-induced ECM degradation and chondrocyte senescence by suppressing the PI3K/AKT/NF-κB signaling pathway and reducing senop-associated secretory phenotype (SASP) expression (Wang Y. et al., 2022). In addition, pterostilbene (20 mg/kg/3 days for 5 weeks by intraperitoneal injection) protects against cartilage degradation in ACLT + DMM-induced OA rat models (Wang Y. et al., 2022). Luteolin (**12**) at the doses of 10 and 20 μM may suppress MMP-13 and ADAMTS5 expression and stimulate collagen II and aggrecan expression in H_2_O_2_-treated mouse chondrocytes (Zhou et al., 2022). 7,8-dihydroflavone (7,8-DHF, **13**) also suppresses the expression of MMP-1, MMP-3, and MMP-13 by activating the NFR2 signaling in H_2_O_2_-treated mouse chondrocytes (Cai et al., 2019a) (Tables 1, 2). Pre-treatment with engeletin (dihydrokaempferol 3-rhamnoside, **24**) may ameliorate TNFα-induced upregulation of MMP-3/-9 expression and downregulation of collagen II and aggrecan expression in rat chondrocytes (Wang H. et al., 2021) (Tables 1, 2). In ACLT-induced rat OA models, intraarticular injection of engeletin (50 μg/week for 5 weeks) effectively protects against histopathological changes (Wang H. et al., 2021). Iron is an essential trace element in the human body. Iron overload may induce chondrocyte apoptosis and ECM degradation, exacerbating the progression of OA. It has been reported that naringenin (**25**) at the doses of 10 and 20 μM exhibits protective effects against iron overload-induced cartilage damage by activating the NRF2/HO-1 signaling pathway in IL-1β-treated human chondrocytes (Pan et al., 2022).

### Suppression of cell death in OA chondrocytes

Hyperoside (**10**) can decrease ROS production, inhibit Bax, cytochrome c, cleaved caspase-9, and cleaved caspase-3 expression, and increase Bcl-xl expression in IL-1β-treated mouse chondrocytes by activating the NRF2 signaling (Sun et al., 2021). 6-Gingerol (**17**) upregulates the expression of ubiquitin-specific peptidase 49 (USP49), promotes the deubiquitination of Axin, and increases the degradation of *β*-catenin, resulting in the inhibition of Wnt/β-catenin signaling and the attenuation of apoptosis in IL-1β-treated rat chondrocytes (Yang L. et al., 2020). Polydatin (**18**) (25, 50, and 100 μg/mL) may promote chondrocyte survival by improving mitochondrial homeostasis via increasing the expression of Parkin and NRF2 in H_2_O_2_-treated chondrocytes (Kang et al., 2020). Procyanidin B2 (PCB2, **26**) at the doses of 20 and 40 μM can suppress IL-1β-induced apoptosis by mediating the NRF2/Bax/Bcl-2 signaling pathway in rat chondrocytes (Cai et al., 2022). Similarly, Luteolin (**12**) can suppress H_2_O_2_-induced ROS production and cell apoptosis in mouse chondrocytes by activating the NRF2 signaling, as detected by decreased caspase-3 and ssDNA expression (Zhou et al., 2022).

Engeletin (**24**, widely distributed in vegetables and fruits) may suppress ROS generation, maintain mitochondrial membrane potential, decrease Bax and cleaved caspase-3 expression, and increase Bcl-2 expression in TNFα-treated rat chondrocytes (Wang H. et al., 2021). Iron overload can induce mitochondrial dysfunctions, trigger oxidative stress, and promote chondrocyte apoptosis. However, naringenin (**25**, a naturally occurring flavanone in citrus fruits) inhibits iron overload-induced chondrocyte apoptosis (Pan et al., 2022). Similarly, Biochanin A (4′-methoxy-5,7-dihydroxy isoflavone, BCA, **27**, extracted from *Astragali Radix* (*Astragalus membranaceus* (Fisch.) Bunge, *Leguminosae*) can regulate iron homeostasis, suppress oxidative stress, and inhibit iron overload-induced mitochondrial dysfunction by up-regulating the NRF2/system xc-/GPX4 signaling pathway in mouse chondrocytes (He et al., 2022) (Table 2). Delphinidin (**28**, a flavonoid present in red wine and berries) at the dose of 40 μM exhibits protective activity against OA development by suppressing oxidative stress, inhibiting apoptosis, and stimulating autophagy. The possible mechanism might be associated with upregulation of the NRF2/ARE signaling and downregulation of the NF-κB pathway in C28/I2 chondrocytes (Lee et al., 2020). Puerarin **8**) also protects chondrocytes by promoting Beclin-1-dependent autophagy and maintaining homeostasis (Li et al., 2021). Curcumin (**20**) maintains mitochondrial homeostasis, induces mitophagy, and protects chondrocytes by mediating the AMPK/PINK1/Parkin signaling pathway (Jin et al., 2022). Curcumin (**20**) also promotes autophagy and reduces apoptosis by inhibiting the PI3K/AKT/mTOR pathway in TNFα-treated chondrocytes (Han et al., 2021).

Licochalcone A (Lico A, **6**) may protect mouse chondrocytes against LPS-induced pyroptosis by down-regulating the NRF2 signaling, as shown by decreased expression of caspase-1, IL-1β, IL-18, and NLRP3 inflammasome (Yan et al., 2020). Consistently, cardamonin (**11**) suppresses IL-1β-induced chondrocyte apoptosis by inhibiting the activity of NLRP3 inflammasome via activating the NRF2 signaling (Jiang and Cai, 2021). Chlorogenic acid (CGA, **29**), a polyphenolic compound, has anti-oxidative activity. It has been demonstrated that CGA (250 μM) can inhibit the expression of pro-apoptotic markers, such as cleaved caspase-3 and PARP, and stimulate the expression of Bcl-xL by attenuating the NF-κB pathway and enhancing the NRF2/ARE signaling, leading to the amelioration of apoptosis in human C28/I2 cells (Zada et al., 2021). Chicoric acid (CA, **30**), extracted from *Taraxacum mongolicum* Hand (Composite), can suppress the expression of pro-inflammatory cytokines, such as IL-6, IL-12, TNFα, COX-2, PGE2, iNOS, NO, and ECM catabolic factors, such as MMP-13 and ADAMTS-5, and increase the production of collagen II and aggrecan by activating the NRF2/HO-1 signaling and inhibiting the PI3K/AKT and NF-κB pathways in C28/I2 cells (Qu et al., 2022). Pinitol (**31**), an ethanolic ingredient from *Pinaceae*, *Leguminosae*, and *Argyrolobium* family, has shown insulin-like effects. It has been reported that pinitol (5 and 10 μM) can ameliorate TNFα-induced chondrocyte senescence and cell cycle arrest by rescuing the NRF2 signaling pathway in C28/I2 cells (Lou et al., 2020).

### Potential mechanisms of flavonoids in activating NRF2 signaling

Myricetin **2**) may promote NRF2 nuclear translocation and activate the NRF2 signaling in IL-1β-treated human chondrocytes. Ly294002, a PI3K/AKT inhibitor, can block the stimulatory effects of myricetin on the activation of NRF2 signaling (Pan et al., 2019). Icariin (ICA, **23**) and EGCG **7**) may downregulate Keap1 expression, upregulate NRF2 expression, and promote the dissociation of Keap1 from NRF2, exhibiting anti-oxidative and chondroprotective activity (Zuo et al., 2019; Zhu et al., 2022a). Similarly, luteolin (**12**) can interrupt the interaction between Keap1 and NRF2, increase the stability of NRF2, and promote NRF2 nuclear translocation by activating AMPKα1 expression in H_2_O_2_-treated mouse chondrocytes (Zhou et al., 2022). Wogonin (**14**) can directly interact with the Kelch domain of Keap1 protein and interrupt the association of Keap1 with NRF2, leading to the stability of NRF2 (Khan et al., 2017). Isovitexin (**4**) has been reported to interact with NRF2 and promote its nuclear translocation (Hu et al., 2021). Another study reports that Procyanidin B2 (PCB2, **26**) can directly bind to the cavity of NRF2 by forming hydrogen bonds, salt bridge, alkyl, and van der Waals, promoting the stability and nuclear translocation of NRF2 in IL-1β-treated rat chondrocytes (Cai et al., 2022).

### Terpenes and terpenoids activate the NRF2 signaling

Terpenoids are a class of natural compounds made up of molecules with the formula (C_5_H_8_)_n_. The structure of terpenes is constructed by the linkage of isoprene units (C_5_H_8_)_n_. Various terpene compounds can be generated by head-to-tail condensation of C5 building blocks. Regarding the number of linked isoprene units, terpenoids can be divided into hemi-, mono-, sesqui-, di-, sester-, tri-, tetra-, and polyterpenes (Arnesen and Borodina, 2022). Terpenoids are excellent candidates for new drug development, due to their multiple pharmacological properties (Atriya et al., 2022). For example, andrographolide (**32**) is a diterpene from *Andrographis paniculata* Burm. f. (*Acanthaceae*) and has been reported to increase the expression of the NRF2/ARE signaling and decrease the NF-κB, MAPK, and JAK/STAT pathways (Burgos et al., 2020). Recently, terpenoids have been the research focus for the therapeutic management of OA.

Monoterpenes Linalool (**33**), an acyclic monoterpene alcohol from *comfrey* (*Symphytum officinale* L., *Boraginaceae*) and *cruciferous* family, has been demonstrated to suppress IL-1β-induced expression of COX-2/PGE2, iNOS/NO, TNFα, IL-6, MMP-13, and ADAMTS5 and increase the production of collagen II and aggrecan in mouse chondrocytes by activating the NRF2/HO-1 pathway and suppressing the NF-κB pathway (Miao et al., 2022).

Sesquiterpenoids Bilobalide (**34**), a sesquiterpenoid isolated from *Ginkgo biloba* L. (*Ginkgoaceae*), has shown various pharmacological activities, such as anti-inflammatory, anti-oxidative, and anti-apoptosis. In a papain intra-articular rabbit OA model, bilobalide (40 mg/kg and 80 mg/kg by gavage administration) has been shown to decrease the expression of MMP-3 and MMP-13 and improve the biomechanical properties by activating the NRF2/HO-1 signaling pathway (Ma et al., 2022a). In addition, bilobalide may induce autophagy and suppress apoptosis in ATDC5 chondrocytes by activating the AMPK/Sirt1/mTOR signaling (Ma et al., 2022c). Patchouli alcohol (PA, **35**) is also a sesquiterpene from *Pogostemon cablin* (Blanco) Benth. (*Lamiaceae*). It has been reported that PA may ameliorate ECM degradation in D-galactose-induced senescent chondrocytes by stimulating the NRF2/HO-1 signaling pathway in mouse chondrocytes (Chen M. et al., 2022).

Diterpenoids Andrographolide (AP, **32**), a natural antioxidant, has been clinically used for treating inflammatory and cancer diseases. In H_2_O_2_-treated rat chondrocytes, AP may suppress oxidative stress by activating the NRF2/ARE signaling pathway. Specifically, AP can inhibit H_2_O_2_-induced expression of MMP-13 and IL-6 and improve chondrocyte apoptosis (Li et al., 2018). Ginkgolide C (**36**), derived from *Ginkgo biloba* L. (*Ginkgoaceae*), has been demonstrated to inhibit H_2_O_2_-induced apoptosis, oxidative stress, and ECM degradation by activating the NRF2/HO-1 pathway and suppressing the NF-κB pathway in rat chondrocytes (Ma et al., 2022b).

Triterpenoids Nomilin (NOM, **37**) is a triterpenoid isolated from some edible citrus fruits and has demonstrated inflammation-modulatory properties. NOM may alleviate the development of OA by decreasing the expression of pro-inflammatory cytokines, such as COX-2/PGE2, iNOS/NO, TNFα, and IL-6, and suppressing ECM degradation by mediating the NRF2 and NF-κB signaling pathways in mouse chondrocytes (Xue et al., 2020). Limonin (LIM, **38**), isolated from lemons and other citrus fruits, has been demonstrated to inhibit IL-1β-induced generation of pro-inflammatory cytokines, such as COX-2/PGE2, iNOS/NO, TNFα, and IL-6, and the expression of MMP-13 and ADAMTS5 in mouse chondrocytes by mediating the NRF2/HO-1/NF-κB signaling pathway (Jin et al., 2021).

Betulin (**39**), isolated from birch bark, is often used for anti-inflammation treatment. Botulin can decrease IL-1β-induced expression of pro-inflammatory cytokines, such as IL-6, TNFα, COX-2, PGE2, iNOS, and NO, and ECM catabolic factors, such as MMP-13 and ADAMTS-5, by activating the AKT/NRF2 pathway and inhibiting the NF-κB pathway in mouse chondrocytes (Ren et al., 2021). Asiaticoside (ASI, **40**), a triterpenoid saponin isolated from *Centella Asiatica* L. (*Apiaceae*), may ameliorate TBHP-induced chondrocyte apoptosis by stimulating the NRF2/HO-1 signaling pathway. Additionally, ASI enhances the production of aggrecan and collagen II and suppresses the expression of MMP-13 and ADAMTS5, improving the degradation of ECM and the progression of OA (Luo et al., 2022). Hederagenin (**41**), a pentacyclic triterpenoid saponin, has been reported anti-inflammatory and anti-oxidative activities by inhibiting the JAK2/STAT3/MAPK signaling pathway and activating the Keap1-NRF2/HO-1/ROS/Bax/Bcl-2 axis, leading to the suppression of ECM degradation and upregulation of collagen II and aggrecan expression in IL-1β-treated C28/I2 cells (Shen et al., 2022) (Table 3). 18β-Glycyrrhetinic acid (18β-GA, **42**), one of the effective metabolites from *Glycyrrhiza glabra* L. (*Leguminosae*), is widely used for treating inflammatory diseases. 18β-GA may suppress IL-1β-induced expression of pro-inflammatory cytokines, such as IL-6, TNFα, COX-2, PGE2, iNOS, and NO, and ECM catabolic factors, such as MMP-13 and ADAMTS-5, by activating the activity of the NRF2/HO-1 pathway in mouse chondrocytes (Chen B. et al., 2021) (Tables 1, 2). In addition, 18β-GA acts as an inhibitor of connexin 43 (Cx43), which plays a role in mechanotransduction. The fluid flow shear stress (FFSS) can upregulate the expression of Cx43 and PGE2, and 18β-GA may abolish FFSS-induced PGE2 expression (Zhang et al., 2014). Akebia saponin D (ASD, **43**), an effective triterpenoid from Rhizome of *Dipsacus asper* Wall (*Caprifoliaceae*), can suppress inflammatory actions by stimulating the NRF2/HO-1 pathway and inhibiting the NF-κB pathway in mouse chondrocytes, protecting against OA development (Gu et al., 2020) (Table 1).

Tetraterpenoids Lycopene (**44**) is often found in vegetables and fruits and has been demonstrated anti-inflammatory activity against OA development by activating the NRF2/HO-1 pathway and reversing the NF-κB/STAT3 pathway, leading to the decreased degradation of ECM in mouse chondrocytes (Zhan et al., 2021). Lycopene also promotes chondrocyte autophagy and suppresses apoptosis by inhibiting MAPK and PI3K/AKT/NF-κB signaling pathways (Wu et al., 2021). Astaxanthin (AST, **45**), also named marine carotenoid, exhibits remarkable anti-oxidative activity by activating the NRF2/ARE signaling. AST has been demonstrated to suppress IL-1β-induced inflammatory responses, cell apoptosis, and ECM degradation by inhibiting the MAPK/NF-κB signaling and stimulating the NRF2 pathway in mouse chondrocytes (Sun et al., 2019) (Table 1).

### Alkaloids activate the NRF2 signaling

Corynoline (COR, **46**), an alkaloid from *Corydalis bungeana* Turcz. (*Papaveraceae* Juss.), has been reported to inhibit inflammatory responses and suppress the NF-κB signaling. COR may suppress IL-1β-induced expression of pro-inflammatory cytokines and ECM degradation by binding and activating NRF2 activity in mouse chondrocytes (Li et al., 2022) (Table 1). Sinomenine (SIN, **47**), isolated from *Sinomenium acutum* Thunb. (*Menispermaceae*), has demonstrated anti-inflammatory effects. It has been reported that SIN reduces the expression of COX-2, PGE2, iNOS, NO, IL-6, and TNFα, inhibits the production of MMPs and ADAMTS5, and suppresses the degradation of collagen II and aggrecan in mouse chondrocytes by activating the NRF2/HO-1 signaling pathway (Wu et al., 2019). Another study reports that SIN can suppress IL-1β-induced MMPs expression by interrupting the interaction between TRAF6 and TAK1, inhibiting JAK2/STAT3 signaling, and increasing SOCS3 expression in SW1353 cells (Qi et al., 2020). Peiminine (PM, **48**) is a bioactive metabolite from *Fritillaria thunbergii* Miq. (*Liliaceae*), which is widely used to treat various diseases. PM has been shown to decrease the production of COX-2, PGE2, iNOS, NO, IL-6, and TNFα in mouse chondrocytes. In addition, PM can decrease the expression of MMP-13 and ADAMTS5 and increase the production of collagen II and aggrecan by stimulating the NRF2/HO-1 signaling and inhibiting the AKT/NF-κB pathway (Luo et al., 2019).

### Miscellaneous types

Coniferaldehyde (**49**), a food flavoring, is a potential NRF2 activator. Coniferaldehyde may ameliorate H_2_O_2_-induced IL-1, IL-6, MMP-1, and MMP-13 expression by activating the NRF2/HO-1 signaling pathway in mouse chondrocytes (Cai et al., 2021a). Ergosterol (**50**) is isolated from the fungus *Agaricus campestris*. Ergosterol has shown chondroprotective activity by activating the NRF2/HO-1 signaling pathway in mouse chondrocytes (Cai et al., 2021b). Maltol (**51**), an aromatic natural metabolite isolated from red ginseng, has shown various biological effects, including anti-inflammation and anti-oxidative stress. It has been reported that maltol exhibits chondroprotective activity *in vivo* and *in vitro*. Specifically, maltol suppresses the production of pro-inflammatory cytokines and the expression of MMP-13 and ADAMTS5 and increases the expression of collagen II and aggrecan by modulating the NRF2 and NF-κB signaling pathways in mouse chondrocytes (Zhu et al., 2021). Monascin (**52**), an azaphilonoid pigment isolated from *Monascus purpureus*-fermented rice, has demonstrated protectiveness in the development of OA. Specifically, monascin can reduce inflammatory responses and suppress ECM degradation by activating the NRF2 signaling and inhibiting the NF-κB pathway in mouse chondrocytes (Zheng et al., 2018). Plumbagin (4-aphthoquinone, **53**) can be found in *Plumbaginaceae*, *Droseraceae*, and *Ebenceae* family. It has been demonstrated that plumbagin exhibits anti-oxidative stress in H_2_O_2_-treated human chondrocytes by activating the NRF2 signaling and inhibiting the NF-κB pathway (Guo et al., 2017).

### Pharmacological applications of NRF2 activators against OA development

Sulforaphane (SFN, **54**), an isothiocyanate widely found in the *Brassicaceae* family and most abundant in broccoli and 3-day-old broccoli sprout extracts (Yagishita et al., 2019), has demonstrated pharmacological effects, such as anti-inflammation and anti-oxidation. Particularly, the anti-oxidative capacity of SFN is associated with the activation of NRF2/ARE signaling pathway (Lu et al., 2023). SFN (50 μM) can significantly decrease H_2_O_2_-induced chondrocyte apoptosis and cartilage degradation by enhancing the expression of Sirt1 (Chen M. et al., 2021). Further study shows that SFN protects human OA chondrocytes against H_2_O_2_-induced oxidative stress, matrix degradation, and hypertrophic differentiation by activating the Keap1/NRF2 signaling pathways (Yang J. et al., 2020) (Table 3). However, SFN (10 μM) may effectively suppress the expression of MMP-1, MMP-13, ADAMTS4, and ADAMTS5 in a NRF2-independent manner in human chondrocytes and SW1353 cells (Davidson et al., 2013). Another study reports that Sulforadex (SFX-01, 100 mg/kg/day for 3 months by oral administration), a stable synthetic form of SFN, can improve the microarchitecture of mouse bone and the symmetry of gait, without producing significant effects on cartilage lesion severity in STR/Ort OA mice (Javaheri et al., 2017). The effective biological effects of SFN against OA have been demonstrated. To achieve a prolonged and sustained activity, an intra-articular injectable SFN-FLGA microsphere system has been designed. SFN-FLGA may significantly decrease inflammatory responses, as indicated by decreased expression of COX-2, MMP-2, and ADAMTS5 in human OA chondrocytes (Ko et al., 2013). Two preparations for SFN have been developed as Sulforadex (SFX-01, 300 mg twice/day for 28 days) and Prostaphane (200 μM once/day for 30 days), which are employed in clinical trials in subarachnoid haemorrhage and prostate cancer patients, respectively (Zolnourian et al., 2020; Dinkova-Kostova and Copple, 2023).

A series of synthetic oleanane triterpenoids has been explored due to their broad applications in preventing and treating chronic diseases. Several potential signaling pathways, such as Keap1/NRF2, PPARγ, JAK/STAT, and PI3K/AKT, have been implicated in the molecular mechanisms of oleanane triterpenoids in mediating their biological actions (Liby and Sporn, 2012). For example, synthetic triterpenoid 2-cyano-3,12-dioxooleanna-1,9 (11)-dien-28-oic acid (CDDO) has been reported to directly interact with the thiol groups of Keap1 and activate the NRF2/ARE signaling pathway (Dinkova-Kostova et al., 2005). Omaveloxolone (RTA408, **55**) is a semisynthetic triterpenoid derived from oleanolic acid and has been considered one of the most potent activators of the NRF2/ARE signaling pathway. RTA408 has been demonstrated to inhibit radiation-induced oxidative stress by disrupting the interaction between Keap1 and NRF2 (Goldman et al., 2015). RTA408 is also effective against inflammation by suppressing the NF-κB signaling pathway (Zhang et al., 2021). RTA408 has been reported to promote chondrocyte proliferation, suppress inflammation and oxidative stress, inhibit chondrocyte apoptosis, and ameliorate ECM degradation by activating the NRF2/ARE signaling and inhibiting the NF-κB pathway in IL-1β-treated chondrocytes. In addition, RTA408 significantly enhances the levels of collagen II and aggrecan, decreases the scores of Osteoarthritis Research Society International (OARSI) and modified Mankin, and improves the microarchitecture of subchondral bone. In addition, RTA408 at doses of 200 μg/kg and 500 μg/kg has been proven to be safe after 8 weeks of treatment in rats (Jiang et al., 2022). The safety of RTA408 (2.5 mg once/day for 28 days/cycle, up to 12 cycles) has been reported in a phase-I clinical trial in patients with metastatic non-small-cell lung cancer or melanoma, and similar assays of RTA408 (150 mg once/day for 48 weeks) in a phase-Ⅱ clinical trial in Friedreich ataxia (Lynch et al., 2019; Madsen et al., 2020).

Dimethyl fumarate (DMF, Tecfidera, **56**), a methyl ester of fumaric acid, is currently the only agent in clinical practice as a NRF2 activator. Notably, DMF increases the activity of mitochondrial tricarboxylic acid (TCA) cycle and the production of ATP. Additionally, DMF and its metabolite monomethyl fumarate have good bioavailability (Sheikh et al., 2013) and exhibit beneficial effects against inflammation and oxidative stress (Kourakis et al., 2020). Mechanically, DMF may stimulate the NRF2/ARE signaling pathway and interact with the anti-inflammatory hydroxycarboxylic acid receptor 2 (HCAR2) (Chen H. et al., 2014). DMF has been approved by FDA as a medication for the therapeutic management of multiple sclerosis. In TNFα-treated human primary chondrocytes, DMF the doses of 1, 5, and 10 μM may inhibit the expression of MMP-1/-3/-13 by down-regulating the JAK/STAT3 signaling pathway, ameliorating the degradation of collagen II, and protecting joint cartilage (Li et al., 2014). ERK1, but not ERK2, plays a critical role in the expression of collagen II and aggrecan by mediating the TGFβ1/Smad3/TIMP-3 axis (Zhu et al., 2017). It has been reported that mice with ERK1 deficiency are susceptible to age-associated OA, due to decreased activity of NRF2. DMF exhibits protective effects against OA in ERK1-knockout mice by stimulating the expression of NRF2 (Chen J. et al., 2022).

Many clinical trials for NRF2 activators, such as plant extracts, dietary supplements, and pure compounds, have been performed and can be found on Clinicaltrials.gov. The drug development of NRF2 activators has been advanced. Upregulation of the NRF2/ARE signaling pathway may provide beneficial effects in adverse environments. It should be noted that over-activation of the NRF2/ARE signaling pathway also has some potential negative effects. Various challenges, such as target specificity, pharmacodynamic responses, short-term and long-term safety considerations, and the variation of NRF2 activity, may adversely affect the expression of NRF2 (Dinkova-Kostova and Copple, 2023). For example, protein-protein interaction (PPI) inhibitors are designed to target Keap1. However, NRF2 is not the only binding partner of Keap1. Inhibition of Keap1 also induces the expression of p62, which may further increase the activation of NRF2. The biological consequences are still needed for detailed investigation (Saito et al., 2016). In addition, NRF2 may promote the expression of the multidrug-resistant protein 3 (MRP3), which acts to export various cytostatic drugs (Slocum and Kensler, 2011; Marchan and Bolt, 2013). ROS at low concentrations can be healthy, and the NRF2/ARE signaling should be functioning properly. For example, ROS are essential to regulate insulin signal transduction and glucose-mediated insulin secretion in pancreatic *β* cells. It can be postulated that over-activation of the NRF2/ARE signaling pathway may be associated with insulin resistance and dysregulated lipid accumulation. In addition, some oxidative modifications of proteins are important for the processes of ubiquitination and protein degradation (Chen J. et al., 2014; Smith et al., 2016). Insulin may increase the production of collagen II and proteoglycan and decrease the breakdown of cartilage (Bradley, 2021). Thus, there is a risk that over-activation of the NRF2/ARE signaling pathway may worsen the pathological changes of OA. Thus, it should be careful to administer some dietary supplements, such as green tea extract and purified EGCG, which may produce over-activation of the NRF2/ARE signaling pathway (Huang et al., 2015).

## Conclusion

**FIGURE 2:**
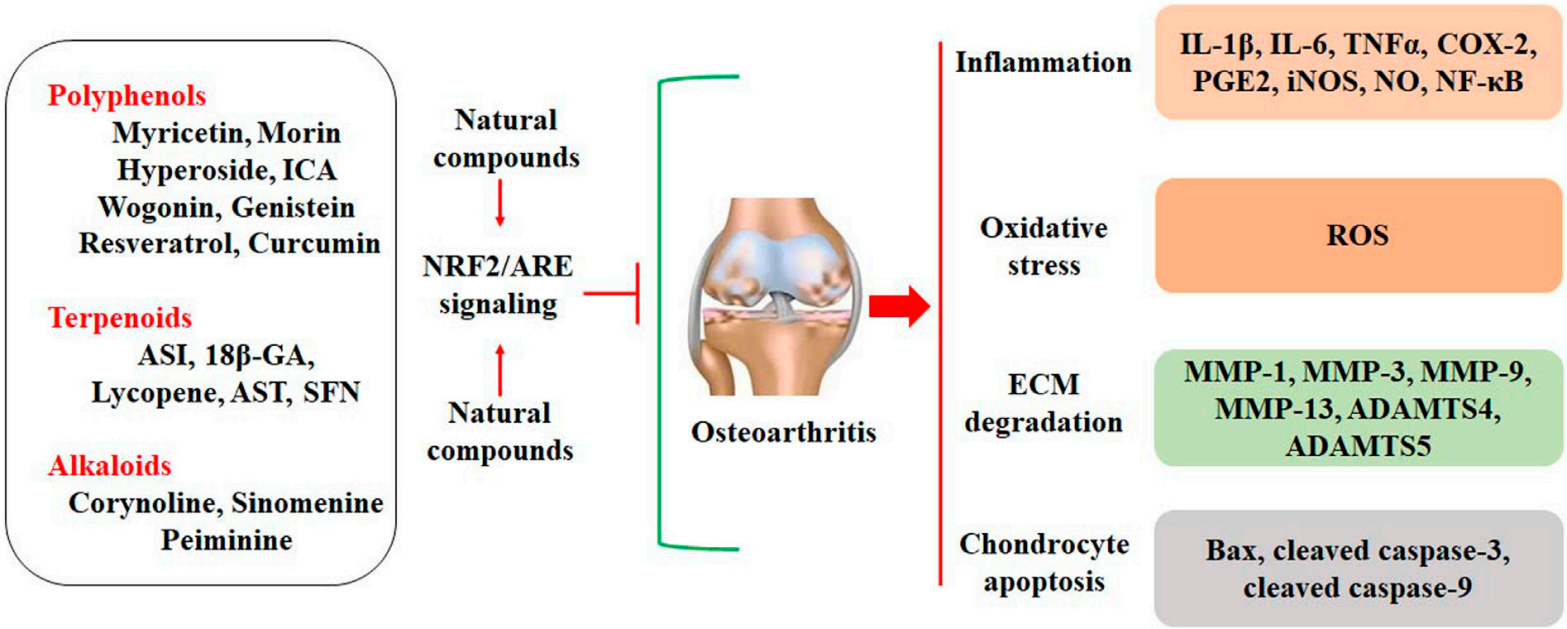
Natural compounds activate the NRF2/ARE signaling to protect against OA development, which is characterized by inflammation, oxidative stress, ECM degradation, and chondrocyte apoptosis.

In this review article, we comprehensively discuss the chondroprotective effects of natural compounds by activating the NRF2/ARE signaling pathway (Figure 2). These effective compounds mainly involve polyphenols, terpenoids, and alkaloids, and they inhibit inflammatory responses, ECM degradation, and chondrocyte apoptosis in OA cartilage. Activation of the NRF2/ARE signaling shows great potential for the therapeutic management of OA. However, we did not discuss the pharmacological properties of these compounds. The information about how these compounds activate the expression of NRF2 is relatively limited. The structure-activity relationship of polyphenols (flavonoids) or terpenoids in activating the NRF2 signaling pathway should be elucidated. The delivery of natural effective compounds to the damaged joint cartilage has become a major therapeutic limitation. In addition, randomized clinical trials of natural compounds against OA development are still required to fully elucidate the chondroprotective effects of natural compounds. These critical limitations should be scientifically addressed in future research.

## References

[B1] Abusarah J. Benabdoune H. Shi Q. Lussier B. Martel-Pelletier J. Malo M. (2017). Elucidating the role of protandim and 6-gingerol in protection against osteoarthritis. J. Cell Biochem. 118 (5), 1003–1013. 10.1002/jcb.25659 27463229

[B2] Agraharam G. Girigoswami A. Girigoswami K. (2022). Myricetin: A multifunctional flavonol in biomedicine. Curr. Pharmacol. Rep. 8 (1), 48–61. 10.1007/s40495-021-00269-2 35036292 PMC8743163

[B3] Akhtar N. Haqqi T. M. (2011). Epigallocatechin-3-gallate suppresses the global interleukin-1beta-induced inflammatory response in human chondrocytes. Arthritis Res. Ther. 13 (3), R93. 10.1186/ar3368 21682898 PMC3218908

[B4] Altay M. A. Ertürk C. Bilge A. Yaptı M. Levent A. Aksoy N. (2015). Evaluation of prolidase activity and oxidative status in patients with knee osteoarthritis: Relationships with radiographic severity and clinical parameters. Rheumatol. Int. 35 (10), 1725–1731. 10.1007/s00296-015-3290-5 25994092

[B5] Arnesen J. A. Borodina I. (2022). Engineering of Yarrowia lipolytica for terpenoid production. Metab. Eng. Commun. 15, e00213. 10.1016/j.mec.2022.e00213 36387772 PMC9663531

[B6] Ashrafizadeh M. Fekri H. S. Ahmadi Z. Farkhondeh T. Samarghandian S. (2020). Therapeutic and biological activities of berberine: The involvement of Nrf2 signaling pathway. J. Cell Biochem. 121 (2), 1575–1585. 10.1002/jcb.29392 31609017

[B7] Atriya A. Majee C. Mazumder R. Choudhary A. N. Mazumder A. Dahiya A. (2022). Insight into the various approaches for the enhancement of bioavailability and pharmacological potency of terpenoids: A review. Curr. Pharm. Biotechnol. 10.2174/1389201024666221130163116 36453488

[B8] Baird L. Yamamoto M. (2020). The molecular mechanisms regulating the KEAP1-NRF2 pathway. Mol. Cell Biol. 40 (13), e00099–20. 10.1128/mcb.00099-20 32284348 PMC7296212

[B9] Braaten J. A. Banovetz M. T. DePhillipo N. N. Familiari F. Russo R. Kennedy N. I. (2022). Biomarkers for osteoarthritis diseases. Life (Basel) 12 (11), 1799. 10.3390/life12111799 36362955 PMC9697481

[B10] Bradley D. (2021). The intriguing intersection of type 2 diabetes, obesity-related insulin resistance, and osteoarthritis. J. Clin. Endocrinol. Metab. 106 (5), e2370–e2372. 10.1210/clinem/dgab009 33511986 PMC8063230

[B11] Burgos R. A. Alarcón P. Quiroga J. Manosalva C. Hancke J. (2020). Andrographolide, an anti-inflammatory multitarget drug: All roads lead to cellular metabolism. Molecules 26 (1), 5. 10.3390/molecules26010005 33374961 PMC7792620

[B12] Cai D. Feng W. Liu J. Jiang L. Chen S. Yuan T. (2019a). 7,8-Dihydroxyflavone activates Nrf2/HO-1 signaling pathways and protects against osteoarthritis. Exp. Ther. Med. 18 (3), 1677–1684. 10.3892/etm.2019.7745 31410125 PMC6676087

[B13] Cai D. Huff T. W. Liu J. Yuan T. Wei Z. Qin J. (2019b). Alleviation of cartilage destruction by sinapic acid in experimental osteoarthritis. Biomed. Res. Int. 2019, 5689613. 10.1155/2019/5689613 30931327 PMC6413400

[B14] Cai D. Wang J. Chen S. Jiang L. Chen J. Wu J. (2021a). Coniferaldehyde prevents articular cartilage destruction in a murine model via Nrf2/HO-1 pathway. Mol. Med. Rep. 23 (3), 224. 10.3892/mmr.2021.11863 33495836 PMC7851827

[B15] Cai D. Yan H. Liu J. Chen S. Jiang L. Wang X. (2021b). Ergosterol limits osteoarthritis development and progression through activation of Nrf2 signaling. Exp. Ther. Med. 21 (3), 194. 10.3892/etm.2021.9627 33488803 PMC7812583

[B16] Cai W. Zhang Y. Jin W. Wei S. Chen J. Zhong C. (2022). Procyanidin B2 ameliorates the progression of osteoarthritis: An *in vitro* and *in vivo* study. Int. Immunopharmacol. 113, 109336. 10.1016/j.intimp.2022.109336 36274486

[B17] Chen B. Ning K. Sun M. L. Zhang X. A. (2023). Regulation and therapy, the role of JAK2/STAT3 signaling pathway in OA: A systematic review. Cell Commun. Signal 21, 67. (1478-811X (Electronic)). 10.1186/s12964-023-01094-4 37013568 PMC10071628

[B18] Chen B. Zhu D. Xie C. Shi Y. Ni L. Zhang H. (2021a). 18β-Glycyrrhetinic acid inhibits IL-1β-induced inflammatory response in mouse chondrocytes and prevents osteoarthritic progression by activating Nrf2. Food Funct. 12 (18), 8399–8410. 10.1039/d1fo01379c 34369548

[B19] Chen C. G. Thuillier D. Chin E. N. Alliston T. (2012). Chondrocyte-intrinsic Smad3 represses Runx2-inducible matrix metalloproteinase 13 expression to maintain articular cartilage and prevent osteoarthritis. Arthritis Rheum. 64 (10), 3278–3289. 10.1002/art.34566 22674505 PMC3544176

[B20] Chen H. Assmann J. C. Krenz A. Rahman M. Grimm M. Karsten C. M. (2014a). Hydroxycarboxylic acid receptor 2 mediates dimethyl fumarate's protective effect in EAE. J. Clin. Invest. 124 (5), 2188–2192. 10.1172/jci72151 24691444 PMC4001545

[B21] Chen J. Chen Z. Yuan P. Huang H. Wang J. Shi P. (2022a). ERK1 loss accelerates the progression of osteoarthritis in aged mice via NRF2/BACH1 signaling. Biochem. Biophys. Res. Commun. 622, 129–135. 10.1016/j.bbrc.2022.07.012 35849954

[B22] Chen J. Zhang Z. Cai L. (2014b). Diabetic cardiomyopathy and its prevention by nrf2: Current status. Diabetes Metab. J. 38 (5), 337–345. 10.4093/dmj.2014.38.5.337 25349820 PMC4209347

[B23] Chen M. Huang L. Lv Y. Li L. Dong Q. (2021b). Sulforaphane protects against oxidative stress-induced apoptosis via activating SIRT1 in mouse osteoarthritis. Mol. Med. Rep. 24 (2), 612. 10.3892/mmr.2021.12251 34184072 PMC8258469

[B24] Chen M. Wen H. Zhou S. Yan X. Li H. (2022b). Patchouli alcohol inhibits D-gal induced oxidative stress and ameliorates the quality of aging cartilage via activating the Nrf2/HO-1 pathway in mice. Oxid. Med. Cell Longev. 2022, 6821170. 10.1155/2022/6821170 35720186 PMC9200550

[B25] Chen W. P. Hu Z. N. Jin L. B. Wu L. D. (2017). Licochalcone A inhibits MMPs and ADAMTSs via the NF-κB and wnt/β-catenin signaling pathways in rat chondrocytes. Cell Physiol. Biochem. 43 (3), 937–944. 10.1159/000481645 28957807

[B26] Chen X. Huang C. Sun H. Hong H. Jin J. Bei C. (2021c). Puerarin suppresses inflammation and ECM degradation through Nrf2/HO-1 axis in chondrocytes and alleviates pain symptom in osteoarthritic mice. Food Funct. 12 (5), 2075–2089. 10.1039/d0fo03076g 33543180

[B27] Chen Z. Zhong H. Wei J. Lin S. Zong Z. Gong F. (2019). Inhibition of Nrf2/HO-1 signaling leads to increased activation of the NLRP3 inflammasome in osteoarthritis. Arthritis Res. Ther. 21 (1), 300. 10.1186/s13075-019-2085-6 31870428 PMC6929452

[B28] Cheng J. Li M. Bai R. (2022). The Wnt signaling cascade in the pathogenesis of osteoarthritis and related promising treatment strategies. Front. Physiol. 13, 954454. 10.3389/fphys.2022.954454 36117702 PMC9479192

[B29] Chistiakov D. A. Sobenin I. A. Revin V. V. Orekhov A. N. Bobryshev Y. V. (2014). Mitochondrial aging and age-related dysfunction of mitochondria. Biomed. Res. Int. 2014, 238463. 10.1155/2014/238463 24818134 PMC4003832

[B30] Collins J. A. Wood S. T. Nelson K. J. Rowe M. A. Carlson C. S. Chubinskaya S. (2016). Oxidative stress promotes peroxiredoxin hyperoxidation and attenuates pro-survival signaling in aging chondrocytes. J. Biol. Chem. 291 (13), 6641–6654. 10.1074/jbc.M115.693523 26797130 PMC4807251

[B31] Dai Y. Liu S. Li J. Li J. Lan Y. Nie H. (2020). SIRT4 suppresses the inflammatory response and oxidative stress in osteoarthritis. Am. J. Transl. Res. 12 (5), 1965–1975. 32509191 PMC7270038

[B32] Davidson R. K. Jupp O. de Ferrars R. Kay C. D. Culley K. L. Norton R. (2013). Sulforaphane represses matrix-degrading proteases and protects cartilage from destruction *in vitro* and *in vivo* . Arthritis Rheum. 65 (12), 3130–3140. 10.1002/art.38133 23983046 PMC4240673

[B33] DelCarlo M. Loeser R. F. (2006). Chondrocyte cell death mediated by reactive oxygen species-dependent activation of PKC-betaI. Am. J. Physiol. Cell Physiol. 290 (3), C802–C811. 10.1152/ajpcell.00214.2005 16236825 PMC1482466

[B34] Dinkova-Kostova A. T. Copple I. M. (2023). Advances and challenges in therapeutic targeting of NRF2. Trends Pharmacol. Sci. 44, 137–149. 10.1016/j.tips.2022.12.003 36628798

[B35] Dinkova-Kostova A. T. Kostov R. V. Kazantsev A. G. (2018). The role of Nrf2 signaling in counteracting neurodegenerative diseases. Febs J. 285 (19), 3576–3590. 10.1111/febs.14379 29323772 PMC6221096

[B36] Dinkova-Kostova A. T. Liby K. T. Stephenson K. K. Holtzclaw W. D. Gao X. Suh N. (2005). Extremely potent triterpenoid inducers of the phase 2 response: Correlations of protection against oxidant and inflammatory stress. Proc. Natl. Acad. Sci. U. S. A. 102 (12), 4584–4589. 10.1073/pnas.0500815102 15767573 PMC555528

[B37] Dong P. F. Jin C. Lian C. Y. Wang L. Wang Z. Y. (2022). Enhanced extracellular matrix degradation in growth plate contributes to manganese deficiency-induced tibial dyschondroplasia in broiler chicks. Biol. Trace Elem. Res. 200 (7), 3326–3335. 10.1007/s12011-021-02921-w 34546491

[B38] Ertürk C. Altay M. A. Selek S. Koçyiğit A. (2012). Paraoxonase-1 activity and oxidative status in patients with knee osteoarthritis and their relationship with radiological and clinical parameters. Scand. J. Clin. Lab. Invest. 72 (5), 433–439. 10.3109/00365513.2012.687116 22616668

[B39] Felson D. T. Lawrence R. C. Dieppe P. A. Hirsch R. Helmick C. G. Jordan J. M. (2000). Osteoarthritis: New insights. Part 1: The disease and its risk factors. Ann. Intern Med. 133 (8), 635–646. 10.7326/0003-4819-133-8-200010170-00016 11033593

[B40] Ghitti E. Rolli E. Crotti E. Borin S. (2022). Flavonoids are intra- and inter-kingdom modulator signals. Microorganisms 10 (12), 2479. 10.3390/microorganisms10122479 36557733 PMC9781135

[B41] Goldman D. C. Alexeev V. Lash E. Guha C. Rodeck U. Fleming W. H. (2015). The triterpenoid RTA 408 is a robust mitigator of hematopoietic acute radiation syndrome in mice. Radiat. Res. 183 (3), 338–344. 10.1667/rr13900.1 25738896 PMC5826655

[B42] Grange L. Nguyen M. V. Lardy B. Derouazi M. Campion Y. Trocme C. (2006). NAD(P)H oxidase activity of Nox4 in chondrocytes is both inducible and involved in collagenase expression. Antioxid. Redox Signal 8 (9-10), 1485–1496. 10.1089/ars.2006.8.1485 16987005

[B43] Gu M. Jin J. Ren C. Chen X. Gao W. Wang X. (2020). Akebia Saponin D suppresses inflammation in chondrocytes via the NRF2/HO-1/NF-κB axis and ameliorates osteoarthritis in mice. Food Funct. 11 (12), 10852–10863. 10.1039/d0fo01909g 33241814

[B44] Guo J. Ma J. Cai K. Chen H. Xie K. Xu B. (2022). Isoflavones from semen sojae preparatum improve atherosclerosis and oxidative stress by modulating Nrf2 signaling pathway through estrogen-like effects. Evid. Based Complement. Altern. Med. 2022, 4242099. 10.1155/2022/4242099 PMC9010186 35432565

[B45] Guo Y. X. Liu L. Yan D. Z. Guo J. P. (2017). Plumbagin prevents osteoarthritis in human chondrocytes through Nrf-2 activation. Mol. Med. Rep. 15 (4), 2333–2338. 10.3892/mmr.2017.6234 28259976

[B46] Han G. Zhang Y. Li H. (2021). The combination treatment of curcumin and probucol protects chondrocytes from TNF-α induced inflammation by enhancing autophagy and reducing apoptosis via the PI3K-Akt-mTOR pathway. Oxid. Med. Cell Longev. 2021, 5558066. 10.1155/2021/5558066 34257809 PMC8249126

[B47] He Q. Yang J. Pan Z. Zhang G. Chen B. Li S. (2022). Biochanin A protects against iron overload associated knee osteoarthritis via regulating iron levels and NRF2/System xc-/GPX4 axis. Biomed. Pharmacother. 157, 113915. 10.1016/j.biopha.2022.113915 36379122

[B48] Henrotin Y. Deby-Dupont G. Deby C. De Bruyn M. Lamy M. Franchimont P. (1993). Production of active oxygen species by isolated human chondrocytes. Br. J. Rheumatol. 32 (7), 562–567. 10.1093/rheumatology/32.7.562 8393361

[B49] Hu X. Li R. Sun M. Kong Y. Zhu H. Wang F. (2021). Isovitexin depresses osteoarthritis progression via the Nrf2/NF-κB pathway: An *in vitro* study. J. Inflamm. Res. 14, 1403–1414. 10.2147/jir.S299557 33883918 PMC8053716

[B50] Huang Y. Li W. Su Z. Y. Kong A. N. (2015). The complexity of the Nrf2 pathway: Beyond the antioxidant response. J. Nutr. Biochem. 26 (12), 1401–1413. 10.1016/j.jnutbio.2015.08.001 26419687 PMC4785809

[B51] Hui T. Zhou Y. Wang T. Li J. Zhang S. Liao L. (2018). Activation of β-catenin signaling in aggrecan-expressing cells in temporomandibular joint causes osteoarthritis-like defects. Int. J. Oral Sci. 10 (2), 13. 10.1038/s41368-018-0016-z 29686224 PMC5966811

[B52] Javaheri B. Poulet B. Aljazzar A. de Souza R. Piles M. Hopkinson M. (2017). Stable sulforaphane protects against gait anomalies and modifies bone microarchitecture in the spontaneous STR/Ort model of osteoarthritis. Bone 103, 308–317. 10.1016/j.bone.2017.07.028 28778596 PMC5571892

[B53] Jia T. Qiao J. Guan D. Chen T. (2017). Anti-inflammatory effects of Licochalcone A on IL-1β-stimulated human osteoarthritis chondrocytes. Inflammation 40 (6), 1894–1902. 10.1007/s10753-017-0630-5 28756519

[B54] Jiang C. Luo P. Li X. Liu P. Li Y. Xu J. (2020a). Nrf2/ARE is a key pathway for curcumin-mediated protection of TMJ chondrocytes from oxidative stress and inflammation. Cell Stress Chaperones 25 (3), 395–406. 10.1007/s12192-020-01079-z 32124251 PMC7192998

[B55] Jiang J. Cai M. (2021). Cardamonin inhibited IL-1β induced injury by inhibition of NLRP3 inflammasome via activating nrf2/NQO-1 signaling pathway in chondrocyte. J. Microbiol. Biotechnol. 31 (6), 794–802. 10.4014/jmb.2103.03057 34024891 PMC9705951

[B56] Jiang M. He J. Gu H. Yang Y. Huang Y. Xu X. (2020b). Protective effect of resveratrol on obesity-related osteoarthritis via alleviating JAK2/STAT3 signaling pathway is independent of SOCS3. Toxicol. Appl. Pharmacol. 388, 114871. 10.1016/j.taap.2019.114871 31881177

[B57] Jiang Z. Qi G. Lu W. Wang H. Li D. Chen W. (2022). Omaveloxolone inhibits IL-1β-induced chondrocyte apoptosis through the Nrf2/ARE and NF-κB signalling pathways *in vitro* and attenuates osteoarthritis *in vivo* . Front. Pharmacol. 13, 952950. 10.3389/fphar.2022.952950 36238561 PMC9551575

[B58] Jin J. Lv X. Wang B. Ren C. Jiang J. Chen H. (2021). Limonin inhibits IL-1β-induced inflammation and catabolism in chondrocytes and ameliorates osteoarthritis by activating Nrf2. Oxid. Med. Cell Longev. 2021, 7292512. 10.1155/2021/7292512 34795843 PMC8595032

[B59] Jin Z. Chang B. Wei Y. Yang Y. Zhang H. Liu J. (2022). Curcumin exerts chondroprotective effects against osteoarthritis by promoting AMPK/PINK1/Parkin-mediated mitophagy. Biomed. Pharmacother. 151, 113092. 10.1016/j.biopha.2022.113092 35550528

[B60] Kang L. Liu S. Li J. Tian Y. Xue Y. Liu X. (2020). Parkin and Nrf2 prevent oxidative stress-induced apoptosis in intervertebral endplate chondrocytes via inducing mitophagy and anti-oxidant defenses. Life Sci. 243, 117244. 10.1016/j.lfs.2019.117244 31891721

[B61] Khan H. Ullah H. Martorell M. Valdes S. E. Belwal T. Tejada S. (2021). Flavonoids nanoparticles in cancer: Treatment, prevention and clinical prospects. Semin. Cancer Biol. 69, 200–211. 10.1016/j.semcancer.2019.07.023 31374244

[B62] Khan N. M. Ahmad I. Haqqi T. M. (2018). Nrf2/ARE pathway attenuates oxidative and apoptotic response in human osteoarthritis chondrocytes by activating ERK1/2/ELK1-P70S6K-P90RSK signaling axis. Free Radic. Biol. Med. 116, 159–171. 10.1016/j.freeradbiomed.2018.01.013 29339024 PMC5815915

[B63] Khan N. M. Haseeb A. Ansari M. Y. Devarapalli P. Haynie S. Haqqi T. M. (2017). Wogonin, a plant derived small molecule, exerts potent anti-inflammatory and chondroprotective effects through the activation of ROS/ERK/Nrf2 signaling pathways in human Osteoarthritis chondrocytes. Free Radic. Biol. Med. 106, 288–301. 10.1016/j.freeradbiomed.2017.02.041 28237856 PMC5490997

[B64] Ko J. Y. Choi Y. J. Jeong G. J. Im G. I. (2013). Sulforaphane-PLGA microspheres for the intra-articular treatment of osteoarthritis. Biomaterials 34 (21), 5359–5368. 10.1016/j.biomaterials.2013.03.066 23601658

[B65] Koike M. Nojiri H. Ozawa Y. Watanabe K. Muramatsu Y. Kaneko H. (2015). Mechanical overloading causes mitochondrial superoxide and SOD2 imbalance in chondrocytes resulting in cartilage degeneration. Sci. Rep. 5, 11722. 10.1038/srep11722 26108578 PMC4480010

[B66] Kourakis S. Timpani C. A. de Haan J. B. Gueven N. Fischer D. Rybalka E. (2020). Dimethyl fumarate and its esters: A drug with broad clinical utility? Pharm. (Basel) 13 (10), 306. 10.3390/ph13100306 PMC7602023 33066228

[B67] Lane N. E. Shidara K. Wise B. L. (2017). Osteoarthritis year in review 2016: Clinical. Osteoarthr. Cartil. 25 (2), 209–215. 10.1016/j.joca.2016.09.025 28100423

[B68] Lee D. Y. Park Y. J. Song M. G. Kim D. R. Zada S. Kim D. H. (2020). Cytoprotective effects of delphinidin for human chondrocytes against oxidative stress through activation of autophagy. Antioxidants (Basel) 9 (1), 83. 10.3390/antiox9010083 31963866 PMC7022588

[B69] Lepetsos P. Papavassiliou A. G. (2016). ROS/oxidative stress signaling in osteoarthritis. Biochim. Biophys. Acta 1862 (4), 576–591. 10.1016/j.bbadis.2016.01.003 26769361

[B70] Li B. Jiang T. Liu H. Miao Z. Fang D. Zheng L. (2018). Andrographolide protects chondrocytes from oxidative stress injury by activation of the Keap1-Nrf2-Are signaling pathway. J. Cell Physiol. 234 (1), 561–571. 10.1002/jcp.26769 30071128

[B71] Li G. Rao H. Xu W. (2021). Puerarin plays a protective role in chondrocytes by activating Beclin1-dependent autophagy. Biosci. Biotechnol. Biochem. 85 (3), 621–625. 10.1093/bbb/zbaa078 33624774

[B72] Li S. Shi Y. Zhang S. Li H. Ye Z. Kong J. (2022). Corynoline alleviates osteoarthritis development via the Nrf2/NF-κB pathway. Oxid. Med. Cell Longev. 2022, 2188145. 10.1155/2022/2188145 35941903 PMC9356246

[B73] Li Y. Tang J. Hu Y. (2014). Dimethyl fumarate protection against collagen II degradation. Biochem. Biophys. Res. Commun. 454 (2), 257–261. 10.1016/j.bbrc.2014.10.005 25305493

[B74] Liang C. Xing H. Wang C. Xu X. Hao Y. Qiu B. (2023). Resveratrol improves the progression of osteoarthritis by regulating the SIRT1-FoxO1 pathway-mediated cholesterol metabolism. Mediat. Inflamm. 2023, 2936236. 10.1155/2023/2936236 PMC9833897 36643587

[B75] Liang J. Wang S. Hu J. Hong X. Zhu M. Liu X. (2022). Targeted inhibition of TXNRD1 prevents cartilage extracellular matrix degeneration by activating Nrf2 pathway in osteoarthritis. Biochem. Biophys. Res. Commun. 635, 267–276. 10.1016/j.bbrc.2022.10.059 36308906

[B76] Liao C. R. Wang S. N. Zhu S. Y. Wang Y. Q. Li Z. Z. Liu Z. Y. (2020). Advanced oxidation protein products increase TNF-α and IL-1β expression in chondrocytes via NADPH oxidase 4 and accelerate cartilage degeneration in osteoarthritis progression. Redox Biol. 28, 101306. 10.1016/j.redox.2019.101306 31539804 PMC6812020

[B77] Liby K. T. Sporn M. B. (2012). Synthetic oleanane triterpenoids: Multifunctional drugs with a broad range of applications for prevention and treatment of chronic disease. Pharmacol. Rev. 64 (4), 972–1003. 10.1124/pr.111.004846 22966038 PMC3462991

[B78] Limagne E. Lançon A. Delmas D. Cherkaoui-Malki M. Latruffe N. (2016). Resveratrol interferes with IL1-β-induced pro-inflammatory paracrine interaction between primary chondrocytes and macrophages. Nutrients 8 (5), 280. 10.3390/nu8050280 27187448 PMC4882693

[B79] Liu F. C. Wang C. C. Lu J. W. Lee C. H. Chen S. C. Ho Y. J. (2019). Chondroprotective effects of genistein against osteoarthritis induced joint inflammation. Nutrients 11 (5), 1180. 10.3390/nu11051180 31137797 PMC6566664

[B80] Loeser R. F. (2010). Age-related changes in the musculoskeletal system and the development of osteoarthritis. Clin. Geriatr. Med. 26 (3), 371–386. 10.1016/j.cger.2010.03.002 20699160 PMC2920876

[B81] Lotz M. Loeser R. F. (2012). Effects of aging on articular cartilage homeostasis. Bone 51 (2), 241–248. 10.1016/j.bone.2012.03.023 22487298 PMC3372644

[B82] Lou C. Deng A. Zheng H. Sun G. Zhao H. Li A. (2020). Pinitol suppresses TNF-α-induced chondrocyte senescence. Cytokine 130, 155047. 10.1016/j.cyto.2020.155047 32200264

[B83] Lu J. Ji M. l. Zhang X. j. Wu H. Li Y. Wang C. (2017). Epigenetic silencing of MIR-375 promotes catilage degradation by targeting JAK2/STAT3 signaling pathway in knee osteoarthritis. Osteoarthr. Cartil. 25, S291. 10.1016/j.joca.2017.02.491

[B84] Lu X. Xu G. Lin Z. Song J. Zhang Y. Wang H. (2023). Sulforaphane delays intervertebral disc degeneration by alleviating endoplasmic reticulum stress in nucleus pulposus cells via activating nrf-2/HO-1. Oxid. Med. Cell Longev. 2023, 3626091. 10.1155/2023/3626091 36647429 PMC9840554

[B85] Luo P. Huang Q. Chen S. Wang Y. Dou H. (2022). Asiaticoside ameliorates osteoarthritis progression through activation of Nrf2/HO-1 and inhibition of the NF-κB pathway. Int. Immunopharmacol. 108, 108864. 10.1016/j.intimp.2022.108864 35623293

[B86] Luo Z. Zheng B. Jiang B. Xue X. Xue E. Zhou Y. (2019). Peiminine inhibits the IL-1β induced inflammatory response in mouse articular chondrocytes and ameliorates murine osteoarthritis. Food Funct. 10 (4), 2198–2208. 10.1039/c9fo00307j 30942801

[B87] Lynch D. R. Farmer J. Hauser L. Blair I. A. Wang Q. Q. Mesaros C. (2019). Safety, pharmacodynamics, and potential benefit of omaveloxolone in Friedreich ataxia. Ann. Clin. Transl. Neurol. 6 (1), 15–26. 10.1002/acn3.660 30656180 PMC6331199

[B88] Ma T. Chen H. Ruan H. Lv L. Yu Y. Jia L. (2022a). Natural product, bilobalide, improves joint health in rabbits with osteoarthritis by anti-matrix degradation and antioxidant activities. Front. Vet. Sci. 9, 1034623. 10.3389/fvets.2022.1034623 36337189 PMC9631767

[B89] Ma T. Jia L. Zhao J. Lv L. Yu Y. Ruan H. (2022b). Ginkgolide C slows the progression of osteoarthritis by activating Nrf2/HO-1 and blocking the NF-κB pathway. Front. Pharmacol. 13, 1027553. 10.3389/fphar.2022.1027553 36386227 PMC9651149

[B90] Ma T. Lv L. Yu Y. Jia L. Song X. Xu X. (2022c). Bilobalide exerts anti-inflammatory effects on chondrocytes through the AMPK/SIRT1/mTOR pathway to attenuate ACLT-induced post-traumatic osteoarthritis in rats. Front. Pharmacol. 13, 783506. 10.3389/fphar.2022.783506 35281931 PMC8905364

[B91] Madsen K. L. Buch A. E. Cohen B. H. Falk M. J. Goldsberry A. Goldstein A. (2020). Safety and efficacy of omaveloxolone in patients with mitochondrial myopathy: MOTOR trial. Neurology 94 (7), e687–e698. 10.1212/wnl.0000000000008861 31896620 PMC7176297

[B92] Marchan R. Bolt H. M. (2013). The cytoprotective and the dark side of Nrf2. Arch. Toxicol. 87 (12), 2047–2050. 10.1007/s00204-013-1165-7 24232176

[B93] Miao Z. Dong M. Wang Z. Ma J. Lin Y. Wu Y. (2022). Linalool inhibits the progression of osteoarthritis via the Nrf2/HO-1 signal pathway both *in vitro* and *in vivo* . Int. Immunopharmacol. 113, 109338. 10.1016/j.intimp.2022.109338 36330908

[B94] Ni B. Pei W. Qu Y. Zhang R. Chu X. Wang Y. (2021). MCC950, the NLRP3 inhibitor, protects against cartilage degradation in a mouse model of osteoarthritis. Oxid. Med. Cell Longev. 2021, 4139048. 10.1155/2021/4139048 34777685 PMC8580635

[B95] Okada K. Mori D. Makii Y. Nakamoto H. Murahashi Y. Yano F. (2020). Hypoxia-inducible factor-1 alpha maintains mouse articular cartilage through suppression of NF-κB signaling. Sci. Rep. 10 (1), 5425. 10.1038/s41598-020-62463-4 32214220 PMC7096515

[B96] Pan X. Chen T. Zhang Z. Chen X. Chen C. Chen L. (2019). Activation of Nrf2/HO-1 signal with Myricetin for attenuating ECM degradation in human chondrocytes and ameliorating the murine osteoarthritis. Int. Immunopharmacol. 75, 105742. 10.1016/j.intimp.2019.105742 31325727

[B97] Pan Z. He Q. Zeng J. Li S. Li M. Chen B. (2022). Naringenin protects against iron overload-induced osteoarthritis by suppressing oxidative stress. Phytomedicine 105, 154330. 10.1016/j.phymed.2022.154330 35905566

[B98] Park J. Lee S. Y. (2022). A review of osteoarthritis signaling intervention using small-molecule inhibitors. Med. Baltim. 101 (32), e29501. 10.1097/md.0000000000029501 PMC9371536 35960127

[B99] Peng Y. J. Lu J. W. Lee C. H. Lee H. S. Chu Y. H. Ho Y. J. (2021). Cardamonin attenuates inflammation and oxidative stress in interleukin-1β-stimulated osteoarthritis chondrocyte through the Nrf2 pathway. Antioxidants (Basel) 10 (6), 862. 10.3390/antiox10060862 34072123 PMC8227809

[B100] Qi W. Gu Y. Wang Z. Fan W. (2020). Sinomenine inhibited interleukin-1β-induced matrix metalloproteinases levels via SOCS3 up-regulation in SW1353 cells. Biol. Pharm. Bull. 43 (11), 1643–1652. 10.1248/bpb.b20-00270 32879146

[B101] Qu Y. Shen Y. Teng L. Huang Y. Yang Y. Jian X. (2022). Chicoric acid attenuates tumor necrosis factor-α-induced inflammation and apoptosis via the Nrf2/HO-1, PI3K/AKT and NF-κB signaling pathways in C28/I2 cells and ameliorates the progression of osteoarthritis in a rat model. Int. Immunopharmacol. 111, 109129. 10.1016/j.intimp.2022.109129 35961266

[B102] Qu Y. Wang C. Liu N. Gao C. Liu F. (2018). Morin exhibits anti-inflammatory effects on IL-1β-stimulated human osteoarthritis chondrocytes by activating the Nrf2 signaling pathway. Cell Physiol. Biochem. 51 (4), 1830–1838. 10.1159/000495684 30504721

[B103] Ren C. Jin J. Hu W. Chen Q. Yang J. Wu Y. (2021). Betulin alleviates the inflammatory response in mouse chondrocytes and ameliorates osteoarthritis via AKT/Nrf2/HO-1/NF-κB Axis. Front. Pharmacol. 12, 754038. 10.3389/fphar.2021.754038 34721040 PMC8548689

[B104] Renaudin F. Oudina K. Gerbaix M. McGilligan Subilia M. Paccaud J. Jaquet V. (2023). NADPH oxidase 4 deficiency attenuates experimental osteoarthritis in mice. RMD Open 9 (1), e002856. 10.1136/rmdopen-2022-002856 36810185 PMC9945017

[B105] Retting K. N. Song B. Yoon B. S. Lyons K. M. (2009). BMP canonical Smad signaling through Smad1 and Smad5 is required for endochondral bone formation. Development 136 (7), 1093–1104. 10.1242/dev.029926 19224984 PMC2668702

[B106] Saha S. Buttari B. Profumo E. Tucci P. Saso L. (2021). A perspective on Nrf2 signaling pathway for neuroinflammation: A potential therapeutic target in alzheimer's and Parkinson's diseases. Front. Cell Neurosci. 15, 787258. 10.3389/fncel.2021.787258 35126058 PMC8813964

[B107] Saito T. Ichimura Y. Taguchi K. Suzuki T. Mizushima T. Takagi K. (2016). p62/Sqstm1 promotes malignancy of HCV-positive hepatocellular carcinoma through Nrf2-dependent metabolic reprogramming. Nat. Commun. 7, 12030. 10.1038/ncomms12030 27345495 PMC4931237

[B108] Scanzello C. R. Goldring S. R. (2012). The role of synovitis in osteoarthritis pathogenesis. Bone 51 (2), 249–257. 10.1016/j.bone.2012.02.012 22387238 PMC3372675

[B109] Shao Z. Pan Z. Lin J. Zhao Q. Wang Y. Ni L. (2020). S-allyl cysteine reduces osteoarthritis pathology in the tert-butyl hydroperoxide-treated chondrocytes and the destabilization of the medial meniscus model mice via the Nrf2 signaling pathway. Aging (Albany NY) 12 (19), 19254–19272. 10.18632/aging.103757 33027770 PMC7732291

[B110] Sheikh S. I. Nestorov I. Russell H. O'Gorman J. Huang R. Milne G. L. (2013). Tolerability and pharmacokinetics of delayed-release dimethyl fumarate administered with and without aspirin in healthy volunteers. Clin. Ther. 35 (10), 1582–1594.e1589. 10.1016/j.clinthera.2013.08.009 24139424

[B111] Shen Y. Teng L. Qu Y. Huang Y. Peng Y. Tang M. (2022). Hederagenin suppresses inflammation and cartilage degradation to ameliorate the progression of osteoarthritis: An *in vivo* and *in vitro* study. Inflammation 46, 655–678. 10.1007/s10753-022-01763-5 36348189

[B112] Slocum S. L. Kensler T. W. (2011). Nrf2: Control of sensitivity to carcinogens. Arch. Toxicol. 85 (4), 273–284. 10.1007/s00204-011-0675-4 21369766

[B113] Smith R. E. Tran K. Smith C. C. McDonald M. Shejwalkar P. Hara K. (2016). The role of the Nrf2/ARE antioxidant system in preventing cardiovascular diseases. Diseases 4 (4), 34. 10.3390/diseases4040034 28933413 PMC5456329

[B114] Sun K. Luo J. Jing X. Guo J. Yao X. Hao X. (2019). Astaxanthin protects against osteoarthritis via Nrf2: A guardian of cartilage homeostasis. Aging (Albany NY) 11 (22), 10513–10531. 10.18632/aging.102474 31772142 PMC6914430

[B115] Sun K. Luo J. Jing X. Xiang W. Guo J. Yao X. (2021). Hyperoside ameliorates the progression of osteoarthritis: An *in vitro* and *in vivo* study. Phytomedicine 80, 153387. 10.1016/j.phymed.2020.153387 33130473

[B116] Sun W. Xie W. Huang D. Cui Y. Yue J. He Q. (2022). Caffeic acid phenethyl ester attenuates osteoarthritis progression by activating NRF2/HO-1 and inhibiting the NF-κB signaling pathway. Int. J. Mol. Med. 50 (5), 134. 10.3892/ijmm.2022.5190 36102306 PMC9542543

[B117] Suraweera T. L. Rupasinghe H. P. V. Dellaire G. Xu Z. (2020). Regulation of nrf2/ARE pathway by dietary flavonoids: A friend or foe for cancer management? Antioxidants (Basel) 9 (10), 973. 10.3390/antiox9100973 33050575 PMC7600646

[B118] Tang Q. Feng Z. Tong M. Xu J. Zheng G. Shen L. (2017). Piceatannol inhibits the IL-1β-induced inflammatory response in human osteoarthritic chondrocytes and ameliorates osteoarthritis in mice by activating Nrf2. Food Funct. 8 (11), 3926–3937. 10.1039/c7fo00822h 28933476

[B119] Tang S. Tang Q. Jin J. Zheng G. Xu J. Huang W. (2018). Polydatin inhibits the IL-1β-induced inflammatory response in human osteoarthritic chondrocytes by activating the Nrf2 signaling pathway and ameliorates murine osteoarthritis. Food Funct. 9 (3), 1701–1712. 10.1039/c7fo01555k 29484338

[B120] Turrens J. F. (2003). Mitochondrial formation of reactive oxygen species. J. Physiol. 552 (2), 335–344. 10.1113/jphysiol.2003.049478 14561818 PMC2343396

[B121] van der Kraan P. M. (2022). Inhibition of transforming growth factor-β in osteoarthritis. Discrepancy with reduced TGFβ signaling in normal joints. Osteoarthr. Cartil. Open 4 (1), 100238. 10.1016/j.ocarto.2022.100238 36474474 PMC9718219

[B122] Wang H. Jiang Z. Pang Z. Qi G. Hua B. Yan Z. (2021a). Engeletin protects against TNF-α-induced apoptosis and reactive oxygen species generation in chondrocytes and alleviates osteoarthritis *in vivo* . J. Inflamm. Res. 14, 745–760. 10.2147/jir.S297166 33727849 PMC7955871

[B123] Wang L. He C. (2022). Nrf2-mediated anti-inflammatory polarization of macrophages as therapeutic targets for osteoarthritis. Front. Immunol. 13, 967193. 10.3389/fimmu.2022.967193 36032081 PMC9411667

[B124] Wang L. Shan H. Wang B. Wang N. Zhou Z. Pan C. (2018a). Puerarin attenuates osteoarthritis via upregulating AMP-activated protein kinase/proliferator-activated receptor-γ coactivator-1 signaling pathway in osteoarthritis rats. Pharmacology 102 (3-4), 117–125. 10.1159/000490418 29961054

[B125] Wang P. Ye Y. Yuan W. Tan Y. Zhang S. Meng Q. (2021b). Curcumin exerts a protective effect on murine knee chondrocytes treated with IL-1β through blocking the NF-κB/HIF-2α signaling pathway. Ann. Transl. Med. 9 (11), 940. 10.21037/atm-21-2701 34350255 PMC8263872

[B126] Wang Q. Deng F. Li J. Guo L. Li K. (2023). The long non-coding RNA SNHG1 attenuates chondrocyte apoptosis and inflammation via the miR-195/IKK-α axis. Cell Tissue Bank. 24 (1), 167–180. 10.1007/s10561-022-10019-3 35796880

[B127] Wang Q. Ying L. Wei B. Ji Y. Xu Y. (2022a). Effects of quercetin on apoptosis and extracellular matrix degradation of chondrocytes induced by oxidative stress-mediated pyroptosis. J. Biochem. Mol. Toxicol. 36 (2), e22951. 10.1002/jbt.22951 34791735

[B128] Wang Y. Chen Y. Chen Y. Zhou B. Shan X. Yang G. (2018b). Eriodictyol inhibits IL-1β-induced inflammatory response in human osteoarthritis chondrocytes. Biomed. Pharmacother. 107, 1128–1134. 10.1016/j.biopha.2018.08.103 30257325

[B129] Wang Y. Zhao H. Jia S. Wang Q. Yao W. Yang Y. (2022b). Senomorphic agent pterostilbene ameliorates osteoarthritis through the PI3K/AKT/NF-κB axis: An *in vitro* and *in vivo* study. Am. J. Transl. Res. 14 (8), 5243–5262. 36105068 PMC9452324

[B130] Wei Y. Jia J. Jin X. Tong W. Tian H. (2018). Resveratrol ameliorates inflammatory damage and protects against osteoarthritis in a rat model of osteoarthritis. Mol. Med. Rep. 17 (1), 1493–1498. 10.3892/mmr.2017.8036 29138829 PMC5780088

[B131] Wu Y. Lin Z. Yan Z. Wang Z. Fu X. Yu K. (2019). Sinomenine contributes to the inhibition of the inflammatory response and the improvement of osteoarthritis in mouse-cartilage cells by acting on the Nrf2/HO-1 and NF-κB signaling pathways. Int. Immunopharmacol. 75, 105715. 10.1016/j.intimp.2019.105715 31310911

[B132] Wu Z. Zhang X. Li Z. Wen Z. Lin Y. (2021). Activation of autophagy contributes to the protective effects of lycopene against oxidative stress-induced apoptosis in rat chondrocytes. Phytother. Res. 35 (7), 4032–4045. 10.1002/ptr.7127 33860572

[B133] Xiong L. Bao H. Li S. Gu D. Li Y. Yin Q. (2023). Cerium oxide nanoparticles protect against chondrocytes and cartilage explants from oxidative stress via Nrf2/HO-1 pathway in temporomandibular joint osteoarthritis. Front. Bioeng. Biotechnol. 11, 1076240. 10.3389/fbioe.2023.1076240 36815898 PMC9937079

[B134] Xue E. X. Lin J. P. Zhang Y. Sheng S. R. Liu H. X. Zhou Y. L. (2017). Pterostilbene inhibits inflammation and ROS production in chondrocytes by activating Nrf2 pathway. Oncotarget 8 (26), 41988–42000. 10.18632/oncotarget.16716 28410217 PMC5522043

[B135] Xue X. H. Xue J. X. Hu W. Shi F. L. Yang Y. (2020). Nomilin targets the Keap1-Nrf2 signalling and ameliorates the development of osteoarthritis. J. Cell Mol. Med. 24 (15), 8579–8588. 10.1111/jcmm.15484 32564468 PMC7412705

[B136] Yagishita Y. Fahey J. W. Dinkova-Kostova A. T. Kensler T. W. (2019). Broccoli or sulforaphane: Is it the source or dose that matters? Molecules 24 (19), 3593. 10.3390/molecules24193593 31590459 PMC6804255

[B137] Yan Z. Qi W. Zhan J. Lin Z. Lin J. Xue X. (2020). Activating Nrf2 signalling alleviates osteoarthritis development by inhibiting inflammasome activation. J. Cell Mol. Med. 24 (22), 13046–13057. 10.1111/jcmm.15905 32965793 PMC7701566

[B138] Yang J. Song X. Feng Y. Liu N. Fu Z. Wu J. (2020a). Natural ingredients-derived antioxidants attenuate H(2)O(2)-induced oxidative stress and have chondroprotective effects on human osteoarthritic chondrocytes via Keap1/Nrf2 pathway. Free Radic. Biol. Med. 152, 854–864. 10.1016/j.freeradbiomed.2020.01.185 32014502

[B139] Yang L. Wang Z. Zou C. Mi Y. Tang H. Wu X. (2020b). Ubiquitin-specific protease 49 attenuates IL-1β-induced rat primary chondrocyte apoptosis by facilitating Axin deubiquitination and subsequent Wnt/β-catenin signaling cascade inhibition. Mol. Cell Biochem. 474 (1-2), 263–275. 10.1007/s11010-020-03850-3 32737772

[B140] Yang Q. Shi Y. Jin T. Duan B. Wu S. (2022). Advanced glycation end products induced mitochondrial dysfunction of chondrocytes through repression of ampkα-SIRT1-PGC-1α pathway. Pharmacology 107 (5-6), 298–307. 10.1159/000521720 35240662

[B141] Yang R. Guo Y. Zong S. Ma Z. Wang Z. Zhao J. (2023). Bardoxolone methyl ameliorates osteoarthritis by inhibiting osteoclastogenesis and protecting the extracellular matrix against degradation. Heliyon 9 (2), e13080. 10.1016/j.heliyon.2023.e13080 36798782 PMC9925876

[B142] Yao Q. Wu X. Tao C. Gong W. Chen M. Qu M. (2023). Osteoarthritis: Pathogenic signaling pathways and therapeutic targets. Signal Transduct. Target Ther. 8 (1), 56. 10.1038/s41392-023-01330-w 36737426 PMC9898571

[B143] Yu S. M. Kim S. J. (2015). The thymoquinone-induced production of reactive oxygen species promotes dedifferentiation through the ERK pathway and inflammation through the p38 and PI3K pathways in rabbit articular chondrocytes. Int. J. Mol. Med. 35 (2), 325–332. 10.3892/ijmm.2014.2014 25435376 PMC4292767

[B144] Zada S. Pham T. M. Hwang J. S. Ahmed M. Lai T. H. Elashkar O. (2021). Chlorogenic acid protects human chondrocyte C28/I2 cells from oxidative stress-induced cell death through activation of autophagy. Life Sci. 285, 119968. 10.1016/j.lfs.2021.119968 34543642

[B145] Zhan J. Yan Z. Kong X. Liu J. Lin Z. Qi W. (2021). Lycopene inhibits IL-1β-induced inflammation in mouse chondrocytes and mediates murine osteoarthritis. J. Cell Mol. Med. 25 (7), 3573–3584. 10.1111/jcmm.16443 33751809 PMC8034440

[B146] Zhang J. Zhang H. Y. Zhang M. Qiu Z. Y. Wu Y. P. Callaway D. A. (2014). Connexin43 hemichannels mediate small molecule exchange between chondrocytes and matrix in biomechanically-stimulated temporomandibular joint cartilage. Osteoarthr. Cartil. 22 (6), 822–830. 10.1016/j.joca.2014.03.017 PMC4706739 24704497

[B147] Zhang L. Zhou Q. Zhou C. L. (2021). RTA-408 protects against propofol-induced cognitive impairment in neonatal mice via the activation of Nrf2 and the inhibition of NF-κB p65 nuclear translocation. Brain Behav. 11 (1), e01918. 10.1002/brb3.1918 33295701 PMC7821557

[B148] Zheng G. Zhan Y. Tang Q. Chen T. Zheng F. Wang H. (2018). Monascin inhibits IL-1β induced catabolism in mouse chondrocytes and ameliorates murine osteoarthritis. Food Funct. 9 (3), 1454–1464. 10.1039/c7fo01892d 29473075

[B149] Zhou S. Shi J. Wen H. Xie W. Han X. Li H. (2020). A chondroprotective effect of moracin on IL-1β-induced primary rat chondrocytes and an osteoarthritis rat model through Nrf2/HO-1 and NF-κB axes. Food Funct. 11 (9), 7935–7945. 10.1039/d0fo01496f 32832965

[B150] Zhou Z. Zhang L. Liu Y. Huang C. Xia W. Zhou H. (2022). Luteolin protects chondrocytes from H(2)O(2)-induced oxidative injury and attenuates osteoarthritis progression by activating AMPK-nrf2 signaling. Oxid. Med. Cell Longev. 2022, 5635797. 10.1155/2022/5635797 35154568 PMC8825676

[B151] Zhu D. C. Wang Y. H. Lin J. H. Miao Z. M. Xu J. J. Wu Y. S. (2021). Maltol inhibits the progression of osteoarthritis via the nuclear factor-erythroid 2-related factor-2/heme oxygenase-1 signal pathway *in vitro* and *in vivo* . Food Funct. 12 (3), 1327–1337. 10.1039/d0fo02325f 33443518

[B152] Zhu W. Tang H. Cao L. Zhang J. Li J. Ma D. (2022a). Epigallocatechin-3-O-gallate ameliorates oxidative stress-induced chondrocyte dysfunction and exerts chondroprotective effects via the Keap1/Nrf2/ARE signaling pathway. Chem. Biol. Drug Des. 100 (1), 108–120. 10.1111/cbdd.14056 35426252

[B153] Zhu W. Tang H. Li J. Guedes R. M. Cao L. Guo C. (2022b). Ellagic acid attenuates interleukin-1β-induced oxidative stress and exerts protective effects on chondrocytes through the Kelch-like ECH-associated protein 1 (Keap1)/Nuclear factor erythroid 2-related factor 2 (Nrf2) pathway. Bioengineered 13 (4), 9233–9247. 10.1080/21655979.2022.2059995 35378052 PMC9162011

[B154] Zhu Y. Gu J. Zhu T. Jin C. Hu X. Wang X. (2017). Crosstalk between Smad2/3 and specific isoforms of ERK in TGF-β1-induced TIMP-3 expression in rat chondrocytes. J. Cell Mol. Med. 21 (9), 1781–1790. 10.1111/jcmm.13099 28230313 PMC5571561

[B155] Zolnourian A. H. Franklin S. Galea I. Bulters D. O. (2020). Study protocol for SFX-01 after subarachnoid haemorrhage (SAS): A multicentre randomised double-blinded, placebo controlled trial. BMJ Open 10 (3), e028514. 10.1136/bmjopen-2018-028514 PMC7170552 32217557

[B156] Zuo C. Cao H. Song Y. Gu Z. Huang Y. Yang Y. (2022). Nrf2: An all-rounder in depression. Redox Biol. 58, 102522. 10.1016/j.redox.2022.102522 36335763 PMC9641011

[B157] Zuo S. Zou W. Wu R. M. Yang J. Fan J. N. Zhao X. K. (2019). Icariin alleviates IL-1β-induced matrix degradation by activating the Nrf2/ARE pathway in human chondrocytes. Drug Des. Devel Ther. 13, 3949–3961. 10.2147/dddt.S203094 PMC6876636 31819369

